# Organ-specific regulation of ATP7A abundance is coordinated with systemic copper homeostasis

**DOI:** 10.1038/s41598-017-11961-z

**Published:** 2017-09-20

**Authors:** Haarin Chun, Tracy Catterton, Heejeong Kim, Jaekwon Lee, Byung-Eun Kim

**Affiliations:** 1 Department of Animal and Avian Sciences, University of Maryland, College Park, MD 20742 USA; 2 Biological Sciences Graduate Program, University of Maryland, College Park, MD 20742 USA; 3 Department of Biochemistry and Redox Biology Center, University of Nebraska, Lincoln, NE 68516 USA

## Abstract

Copper (Cu) is an essential cofactor for various enzymatic activities including mitochondrial electron transport, iron mobilization, and peptide hormone maturation. Consequently, Cu dysregulation is associated with fatal neonatal disease, liver and cardiac dysfunction, and anemia. While the Cu transporter ATP7A plays a major role in both intestinal Cu mobilization to the periphery and prevention of Cu over-accumulation, it is unclear how regulation of ATP7A contributes to Cu homeostasis in response to systemic Cu fluctuation. Here we show, using Cu-deficient mouse models, that steady-state levels of ATP7A are lower in peripheral tissues (including the heart, spleen, and liver) under Cu deficiency and that subcutaneous administration of Cu to these animals restore normal ATP7A levels in these tissues. Strikingly, ATP7A in the intestine is regulated in the opposite manner - low systemic Cu increases ATP7A while subcutaneous Cu administration decreases ATP7A suggesting that intestine-specific non-autonomous regulation of ATP7A abundance may serve as a key homeostatic control for Cu export into the circulation. Our results support a systemic model for how a single transporter can be inversely regulated in a tissue-specific manner to maintain organismal Cu homeostasis.

## Introduction

Copper (Cu) is an essential trace element required for a vast array of cellular processes in organisms from bacteria to humans. Cu serves as a redox-reactive, catalytic cofactor in the enzymatic reactions that drive oxidative phosphorylation, iron (Fe) acquisition, protection from oxidative stress, neuropeptide maturation, blood clotting, and angiogenesis^1–3^. However, the ability of Cu to undergo reversible redox changes also makes this element deleterious to the organism when present in excess^4,5^. Because of its dichotomous potential as both an essential cofactor and a toxic agent, cells have evolved sophisticated mechanisms for the regulation of Cu acquisition, storage, and distribution^6,7^. As all organismal Cu must pass through the intestine prior to distribution to other tissues^8,9^, cross-talk must take place among tissue types to ensure that import and export of Cu from the intestine are coordinated with extraintestinal Cu requirements.

Cu is taken up by intestinal epithelial cells (IECs), routed for incorporation into Cu-dependent proteins, and mobilized across the basolateral membrane into peripheral circulation. Ctr1 is a homotrimeric integral membrane protein conserved in eukaryotes ranging from yeast to humans that drives intestinal Cu absorption with high affinity and specificity^10,11^. Once Cu is imported into IECs, the Cu-transporting P-type ATPase known as ATP7A conveys it across the basolateral membrane of the enterocyte, where it is delivered into portal circulation. The ATP7B Cu exporter, which is structurally related to ATP7A, is expressed in the liver and removes excess Cu via transport across the apical membrane into the bile^12^.

In humans, mutations in the ATP7A gene are known to cause Menkes disease^13^, which often leads to early childhood mortality as a consequence of reduced Cu efflux from enterocytes into the bloodstream^14,15^. ATP7A is normally localized to the trans-Golgi network (TGN)^16^ where it is essential for the insertion of Cu into important secreted enzymes such as tyrosinase^17^ and lysyl oxidase^18^. When Cu is abundant, ATP7A-containing vesicles traffic to the plasma membrane for Cu efflux^16^. The Cu-induced trafficking of ATP7A has presumably evolved to allow this transporter to shift its function from metallation of secreted cuproenzymes in the TGN to the cellular-protective export of excess cellular Cu across the plasma membrane, as well as for absorption of dietary Cu by enterocytes in the intestine.

In contrast to other organisms, systemic Cu fluctuations in mammalian cells do not alter expression of Cu homeostasis genes at the transcriptional level^7^. While it is generally accepted that regulation of mammalian Cu homeostasis at the cellular level occurs predominantly via posttranscriptional mechanisms, these mechanisms have not yet been shown to play a role in organism-wide systemic Cu homeostasis. In this study, we present evidence that low Cu levels stimulate a reduction in ATP7A protein abundance in tissue culture and in a whole animal model. Specifically, Cu-deficient mice exhibited substantially reduced ATP7A steady-state protein levels in peripheral tissues, including the heart, spleen, and liver, consistent with results from cell culture data. Compared to peripheral tissues, however, ATP7A expression in the intestine was inversely regulated in response to Cu levels, hinting at a distinct regulatory role for intestinal ATP7A in organismal Cu homeostasis. Our data suggest that this intestine-specific regulation of ATP7A protein levels may serve as a key homeostatic mechanism for control of the efflux of this essential, yet potentially toxic, trace element into circulation, thus providing new molecular insights into how intestinal Cu export is regulated for systemic Cu homeostasis in response to peripheral Cu deficiency.

## Results

### Elevated Cu increases ATP7A protein levels in cultured cells

**Figure 1:**
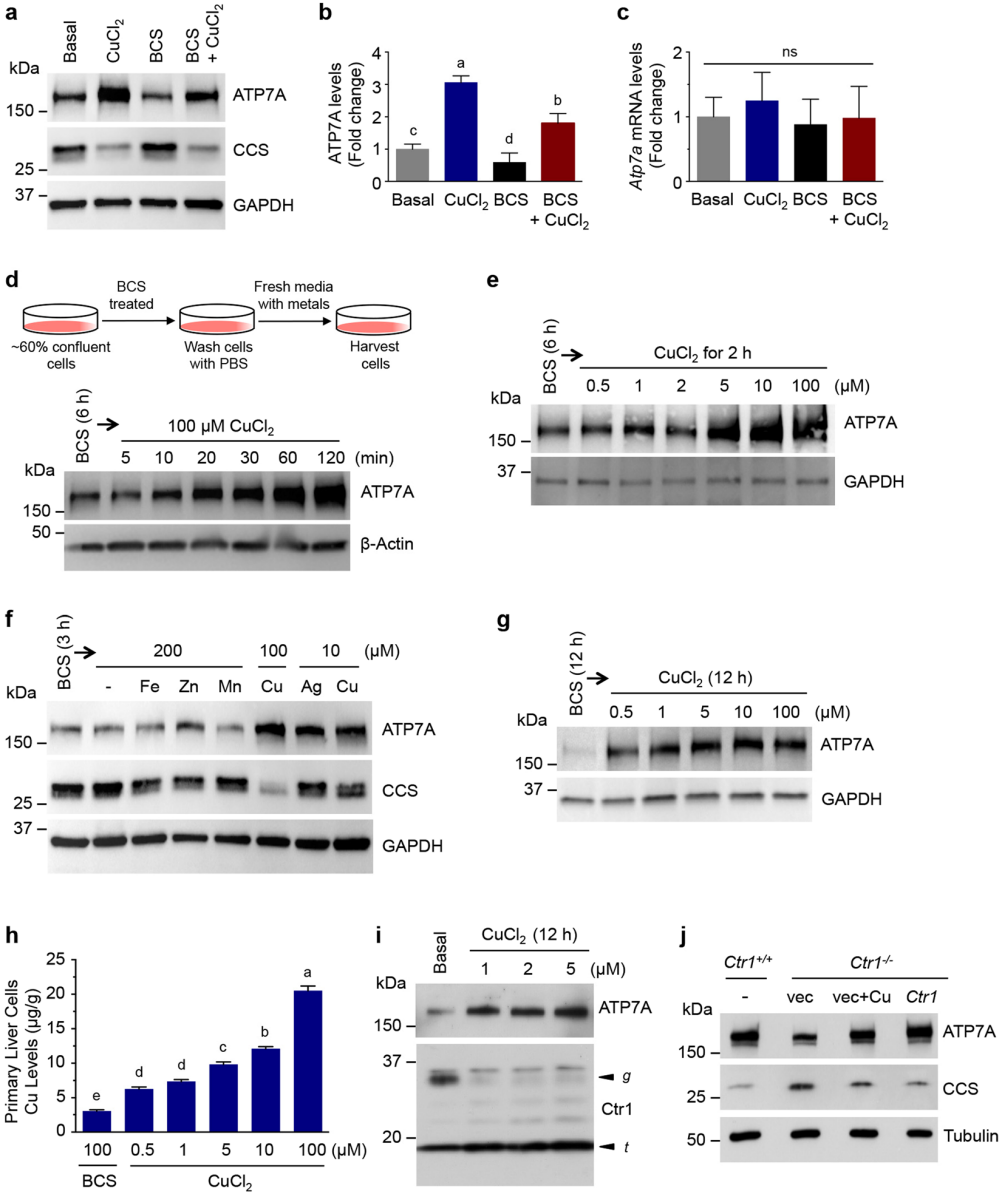
Elevated Cu increases ATP7A protein levels in cultured cells. (**a**) IEC-6 cells were exposed to basal medium, 100 µM CuCl_2_ for 3 h, 300 µM BCS for 6 h, or 300 µM BCS plus 100 µM CuCl_2_ for 6 h. Total protein extracts were probed with an anti-ATP7A antibody, anti-CCS, and anti-GAPDH as a loading control. A representative immunoblot of seven independent experiments is shown here. (**b**,**c**) Relative expression levels of ATP7A protein (**b**) and *Atp7a* mRNA (**c**) in IEC-6 cells exposed to CuCl_2_, BCS, or BCS with CuCl_2_ were normalized to basal media conditions from four independent immunoblot and RT-qPCR experiments. GAPDH protein and *18S rRNA* mRNA, respectively, were used as internal controls in these experiments. Error bars indicate mean ± SD of four independent experiments. Means that do not share a letter are significantly different (*P* < 0.05); ns, not significant (*P* > 0.05) (one-way ANOVA, Tukey’s post hoc test). (**d**,**e**) IEC-6 cells at ~60% confluence were preincubated with 300 µM BCS-treated medium for 6 h to minimize expression levels of ATP7A. Cells were further supplemented with 100 µM CuCl_2_ for the indicated time (**d**) or with the indicated Cu concentrations for 2 h (**e**). β-actin and GAPDH levels shown in the lower panel demonstrate equal protein loading. Data are representative of two (**d**) and three (**e**) independent experiments. (**f**) IEC-6 cells were preincubated with 300 μM BCS for 3 h before switching to media containing 200 µM of FeCl_3_ (Fe), ZnCl_2_ (Zn), MnCl_2_ (Mn), 10 µM of AgNO_3_ (Ag), or 10 or 100 μM of CuCl_2_ (Cu) for 2 h, and 100 μg of protein extracts were subjected to immunoblotting. Reduced levels of CCS indicate increased bioavailable Cu levels, and GAPDH levels are shown to indicate protein loading of samples. Immunoblots are representative results of four independent experiments. (**g**) Mouse primary liver cells treated with Cu supplementation were analyzed by immunoblotting. Mouse liver cells were pretreated for 12 h in BCS-containing medium and then exposed to medium containing indicated concentrations of CuCl_2_ for additional 12 h. GAPDH levels were assayed as a loading control. Immunoblots are representative results of four independent experiments. (**h**) Total Cu levels in mouse primary liver cells were measured by ICP-MS upon supplementation with 100 µM BCS or the indicated Cu concentrations. Data are presented as mean ± SD from four biological replicates. Values with one different letter are significantly different from each other (*P* < 0.05) (One-way ANOVA, Tukey’s post hoc test). **(i)** Immunoblotting of ATP7A and Ctr1 levels in HUVEC cells treated with a range of concentrations of CuCl_2_ for 12 h. The arrowheads labeled *g* and *t* indicate the full-length glycosylated monomer and the amino-terminal truncation forms of Ctr1, respectively. The glycosylated and truncated form of Ctr1 have previously been demonstrated to represent mature glycosylated Ctr1 species, and amino-terminal cleaved truncated Ctr1, respectively^33^. Data are representative of three independent experiments. (**j**) ATP7A protein levels in *Ctr1* ^+/+^ and *Ctr1* ^−/−^ MEFs. Total protein extracts isolated from wild type (*Ctr1* ^+/+^) MEFs, *Ctr1* ^−/−^ MEFs transfected with empty vector (vec) exposed to basal media or 100 μM CuCl_2_ for overnight (vec + Cu), and *Ctr1* ^−/−^ MEFs transfected with a plasmid expressing Ctr1 were resolved by SDS-PAGE and analyzed by immunoblotting. Data are representative for three independent experiments. Full-length blots are presented in Supplementary Figure 11.

To explore Cu-responsive regulation of ATP7A, non-polarized rat intestinal epithelial cells (IEC-6) were grown in basal media and treated with CuCl_2_ (100 µM), the membrane impermeable Cu (I)-specific chelator bathocuproine disulfonic acid (BCS, 300 µM), or 300 µM BCS together with 100 µM CuCl_2_, and analyzed for ATP7A abundance by immunoblotting. A significant elevation in ATP7A protein levels was observed in media containing 100 µM Cu, whereas ATP7A levels were decreased in BCS-treated cells (Fig. 1a ﻿and b). Notably, mRNA levels of *Atp7a* were not altered by either treatment (Fig. 1c). ATP7A levels were enhanced in cells treated simultaneously with BCS and CuCl_2_ indicating that the BCS-induced ATP7A decrease is caused by a Cu-limitation. Increased levels of the Cu chaperone for superoxide dismutase (CCS) observed in BCS-treated cells (Fig. 1a) suggested that available cellular Cu was limited, as CCS is elevated when Cu is scarce^19,20^. ATP7A levels in IEC-6 cells were increased in response to 100 µM CuCl_2_ in a time-dependent manner (Fig. 1d), in agreement with a previous report by the Collins group^21^. Abundance of an ectopically-expressed recombinant-tagged (HA-GFP) ATP7A by Cu and BCS was regulated similarly to endogenous ATP7A (Fig. S1).

To determine the sensitivity of ATP7A elevation under Cu surplus conditions, IEC-6 cells were pre-grown in BCS-treated media for 6 h, washed with phosphate-buffered saline (PBS), and exposed to a range of Cu concentrations for 2 h, followed by assessment of ATP7A levels. Cu levels as low as 1 µM were sufficient to enhance expression of ATP7A as compared to cells in BCS-treated media, and ATP7A levels progressively increased until reaching saturation at treatment with 10 µM of Cu (Fig. 1e), which is within the physiological range of Cu concentrations in plasma^22^.

To test the specificity of ATP7A metal-responsiveness, ATP7A levels were investigated in media containing 200 µM FeCl_3_, ZnCl_2_, MnCl_2_, or 10 µM AgNO_3_; exposure to iron, zinc, and manganese at a 2:1 molar excess over Cu resulted in no substantial increase in ATP7A protein levels (Fig. 1f). However, ATP7A levels were increased by treatment with 10 µM Ag with similar efficiency to equimolar concentrations of Cu. As Ag(I) is isoelectronic to Cu(I), these findings suggest that Cu(I), rather than Cu(II), is the primary driver of this process.

To further explore this phenomenon in primary cells, liver cells were isolated from the livers of C57BL/6 mice at postnatal day 12 (P12), as liver Cu content and ATP7A expression levels in perinatal mice are higher than those in adult mice, suggesting a role for ATP7A in the livers of neonatal mice^23–26^. Isolated primary liver cells were pretreated with BCS overnight and exposed to a range of Cu concentrations. The primary cells treated with as low as 0.5 µM Cu showed significantly increased ATP7A protein abundance (Fig. 1g) demonstrating that increased cellular Cu levels, as shown by ICP-MS analysis (Fig. 1h), are associated with elevated ATP7A expression. Moreover, low micromolar Cu (1 µM) was also sufficient to induce ATP7A expression in human umbilical vein endothelial cell (HUVEC) cultures (Fig. 1i). Degradation of the active, glycosylated full-length form of the Ctr1 Cu importer (with a concomitant increase in the truncated Ctr1 species) was observed upon Cu treatment, as described previously in HEK293 cells^9^. These results demonstrate that cells harbor a mechanism for regulation of ATP7A protein levels to adjust the export of Cu in response to exogenous Cu. Although elevation of ATP7A occurs in response to Cu media supplementation, it is unclear whether extracellular Cu triggers this process indirectly, or whether Cu in the growth media enters cells and elevates intracellular Cu, thereby triggering ATP7A stabilization. In order to clarify the mechanism, we investigated the degradation of ATP7A in mouse embryonic fibroblasts (MEF) lacking the high-affinity Cu importer Ctr1 (*Ctr1* ^−/−^)^27^, along with *Ctr1* ^+/+^ wild type cells, and *Ctr1* ^−/−^ cells transfected with a wild type Ctr1-expressing plasmid, grown in Cu-supplemented media. ATP7A protein levels in *Ctr1* ^−/−^ MEF cells transfected with vector plasmid were lower than those in *Ctr1* ^+/+^ MEFs and *Ctr1* ^−/−^ MEFs treated with exogenous Cu (Fig. 1j), indicating that the intracellular Cu pool modulates ATP7A abundance.

### Post-translational control of ATP7A abundance in response to Cu

**Figure 2:**
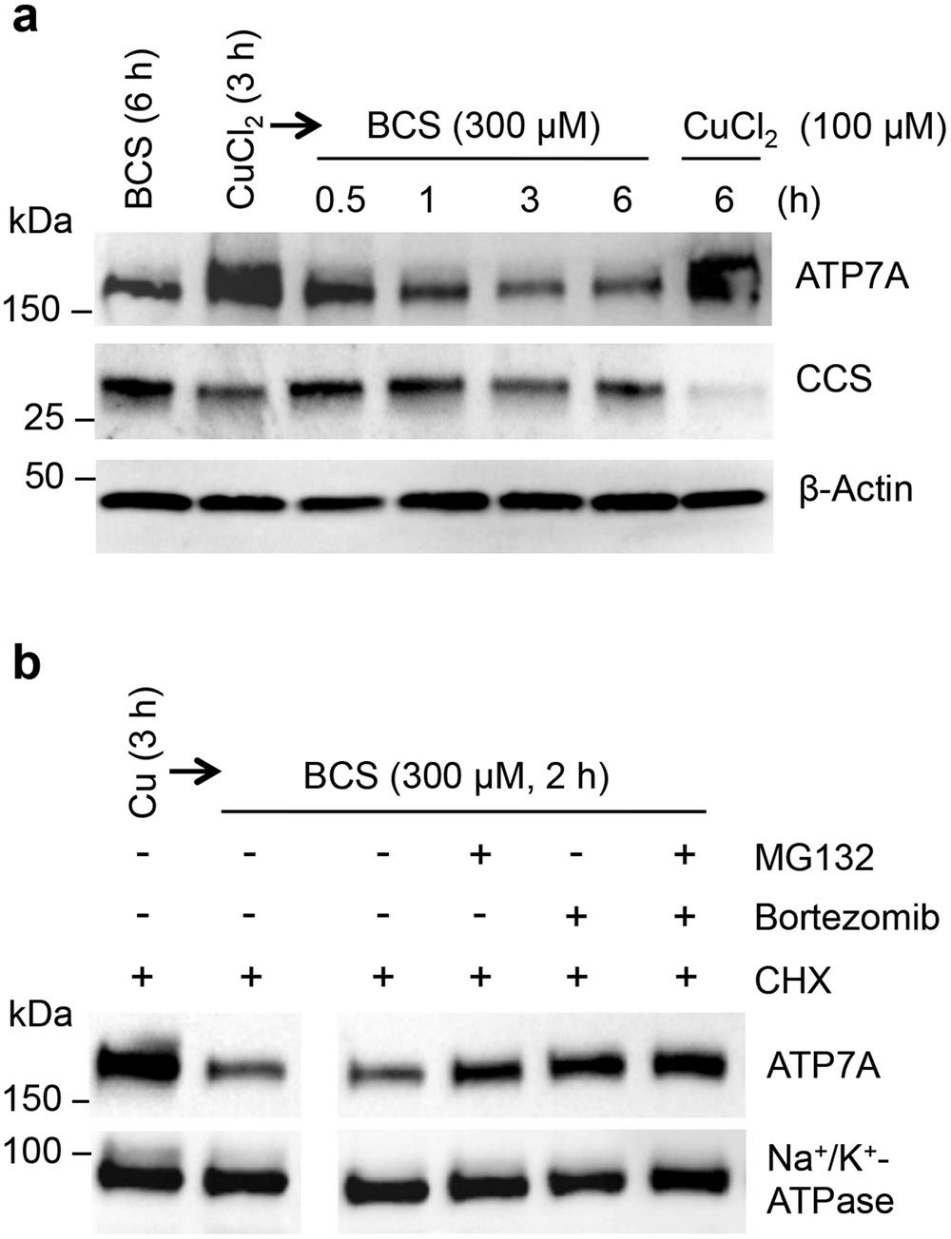
Cu-deficiency stimulates the degradation of ATP7A protein. **(a)** IEC-6 cells pretreated with media containing 100 μM CuCl_2_ for 2.5 h were further treated with 50 μg/mL cycloheximide (CHX). Following 30 min incubation, cells were exposed to fresh culture media containing 300 μM BCS and 50 μg/ml CHX and then lysed after 0.5, 1, 3, or 6 h (lanes 3–6). Whole cell lysates were processed for immunoblotting analysis using antibodies as indicated. Immunoblot images are representative results of four independent experiments. **(b)** IEC-6 cells were pretreated with media containing 100 μM CuCl_2_ for 1 h before addition of 20 μM MG132, 100 nM bortezomib, or DMSO (vehicle control) and treated for another 90 min. Cells were further treated with 50 μg/mL CHX for 30 min, and then the media was replaced with fresh media containing 300 μM BCS and 50 μg/mL CHX for 2 h in the presence of MG132 and/or bortezomib, or DMSO. Na^+^/K^+^-ATPase served as a loading control. Representative immunoblots of four independent experiments are shown. Full-length blots are presented in Supplementary Figure 12.

A previous report showing no changes in * Atp7a* mRNA levels in response to supplemental Cu in rat IECs^21^ suggests steady-state ATP7A protein levels are regulated post-transcriptionally. ATP7A is very stable protein, with a reported half-life of over 40 h in a variety of cells^21,28–30^. To further explore Cu-dependent ATP7A regulation, IEC-6 cells were treated with Cu or BCS in the presence of the translation inhibitor cycloheximide (CHX), and steady-state levels of ATP7A were analyzed by immunoblotting over time. In IEC-6 cells, elevated steady-state levels of ATP7A by Cu treatment were significantly diminished within an hour of exposure to 300 µM BCS; no reduction in ATP7A was observed under Cu-excess conditions upon 100 µM Cu treatment (Fig. 2a).

To test whether this enhanced turnover of ATP7A occurs through the proteasome pathway, IEC-6 cells pretreated with Cu were exposed to BCS along with the proteasome inhibitors MG132 and bortezomib, alone or in combination. These proteasome inhibitors partially abrogated the decrease in steady-state levels of ATP7A protein in response to cellular Cu deprivation (Fig. 2b), suggesting that the reduction in ATP7A protein levels was due, at least in part, to increased proteolysis via a proteasomal pathway.

### A liver-specific role for active Cu sequestration

**Figure 3:**
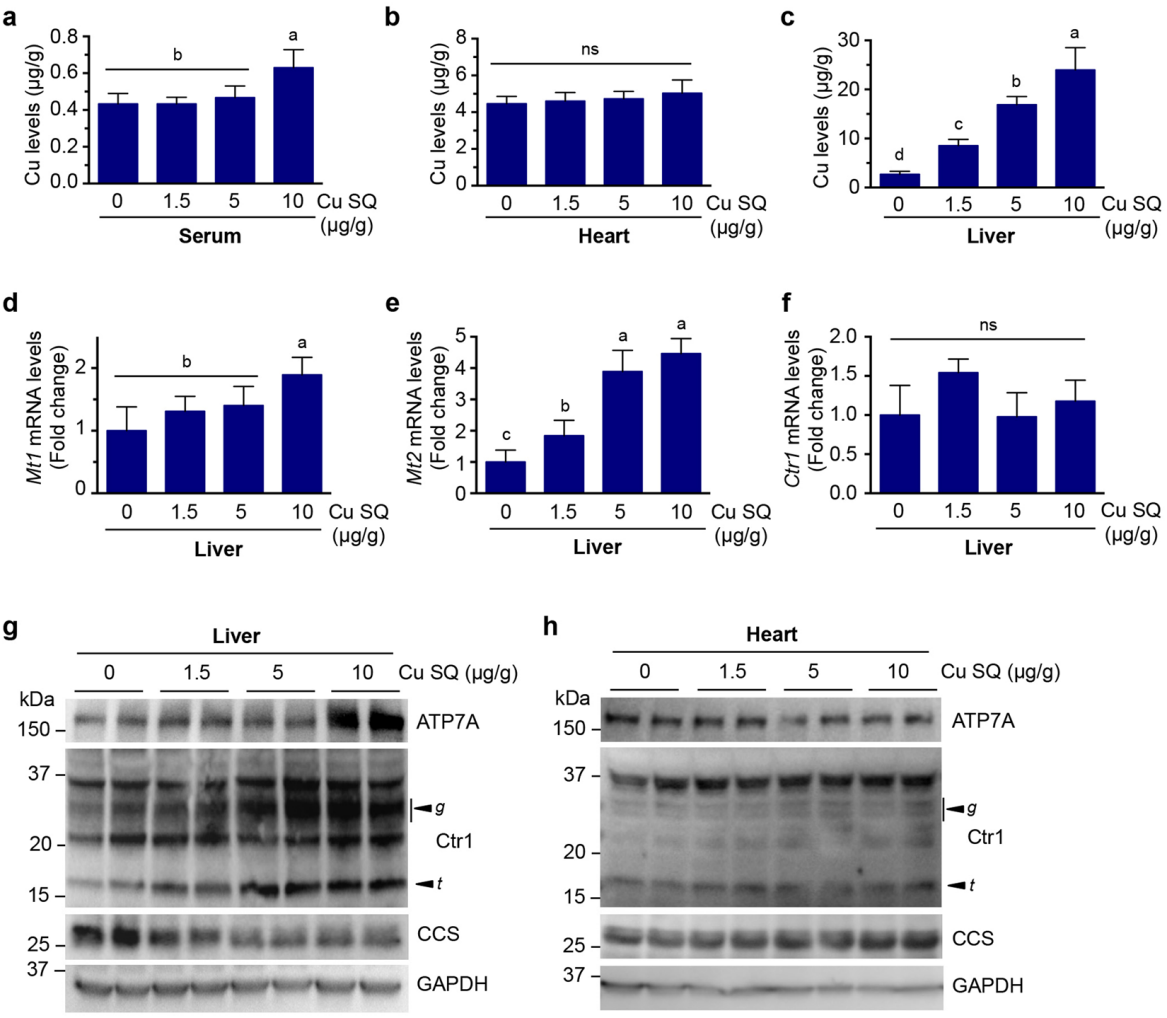
Ctr1 protein levels and Cu accumulation in the liver are elevated by subcutaneous Cu administration. **(a**–**c)** Cu levels measured by ICP-MS in serum (**a**), heart (**b**), and liver (**c**) from C57BL/6 mice (P10) SQ administered indicated amounts of Cu-histidine for three consecutive days beginning at P7. Data (means ± SD) are from seven to nine mice of each condition (n = 2–5 mice for males and females per Cu dose), and means marked with different letter superscripts are significantly different at *p* = 0.05 (one-way ANOVA, Tukey’s post hoc test). **(d**–**f)** Quantitative RT-qPCR analysis of mRNA levels of *Mt1, Mt2, and Ctr1*. Levels of *Mt1* (**d**), *Mt2* (**e**), *Ctr1* (**f**) transcripts were measured relative to *Gapdh* mRNA levels in the livers of individual mice at P10 (male, n = 2; female, n = 2) for each condition, which were SQ administered Cu or saline at P7 for three consecutive days. Bars indicate mean ± SD for each condition. Means that do not share a letter are significantly different (P < 0.05). ns, not significant (*P* > 0.05) (one-way ANOVA, Tukey’s post hoc test). **(g** and **h)** Immunoblot analysis of ATP7A, Ctr1, CCS, and GAPDH in liver (**g**) and heart (**h**) extracts from two representative mice administered saline or indicated amounts of Cu-histidine per body weight (µg/g) for three consecutive days beginning at P7. The arrowheads indicate the glycosylated full-length (*g*) and truncated form (*t*) of Ctr1, respectively. GAPDH levels were assayed as a loading control. Results are representative of three to four independent experiments performed on a total of male (liver, n = 5, 4, 4, and 6; heart, n = 5, 3, 5 and 6) and female mice (liver, n = 6, 4, 4, and 5; heart, n = 6, 4, 2, and 5), which were SQ administered with 0, 1.5, 5, and 10 µg CuCl_2_–histidine per body weight (**g**). Full-length blots are presented in Supplementary Figure 13.

To test the physiological significance of Cu-responsive ATP7A abundance regulation, C57BL/6 mice were subcutaneously (SQ) administered Cu or saline^9^ at P7 for three consecutive days. Mice were dissected at P10 and assayed for Cu accumulation in tissues by ICP-MS. While no marked Cu accumulation was observed in the serum or the heart, the liver showed highly elevated Cu accumulation in a dose-dependent manner (Fig. 3a–c). As metallothionein (MT) genes are induced by high Cu exposure^31^, mRNA levels of *Mt-1* and *Mt-2* genes were assessed to explore whether these correlate with hepatic Cu accumulation. As expected, while expression of *Mt-1* was modestly increased only in mice administered Cu at the concentration of 10 μg/g BW, *Mt-2* gene was significantly elevated in mice administered SQ Cu compared to control mice (Fig. 3d and e). Hepatic ATP7A expression was enhanced only in mice administered Cu at the concentration of 10 μg/g total body weight (BW) (Fig. 3g and S 2a). Elevated serum Cu levels were also detected only in mice treated with 10 μg Cu/g BW (Fig. 3a), suggesting that this level of Cu exceeds the capacity of the liver to excrete surplus Cu.

As the Cu-specific importer Ctr1 is inactivated through endocytosis and degradation in cultured cells in response to Cu treatment (Fig. 1i)^32^, and the mature glycosylated form of Ctr1 was increased in the intestines and hearts of wild type mice fed a Cu-deficient diet^33^, we performed immunoblot analysis of Ctr1 protein levels in the livers of mice administered with Cu. Unexpectedly, whereas mRNA levels of *Ctr1* were not changed (Fig. 3f), the glycosylated full-length form of Ctr1 protein was strongly elevated in the livers of Cu-treated mice, with a concomitant increase in hepatic Cu accumulation and a reduction in CCS levels, in a dose-dependent manner (Fig. 3c,g and S2). However, peripheral tissues such as the heart, spleen and brain showed no obvious changes in protein abundances of Ctr1 and ATP7A (Fig. 3h and S3). These data suggest that Cu-responsive elevation of the mature glycosylated Ctr1 protein is liver-specific, suggesting a mechanism by which Cu can be sequestered in the liver under Cu-excess conditions. Taken together, these findings suggest the existence of a liver-specific role for Ctr1 in active Cu detoxification for systemic Cu homeostasis.

### Organ-specific regulation of ATP7A abundance

**Figure 4:**
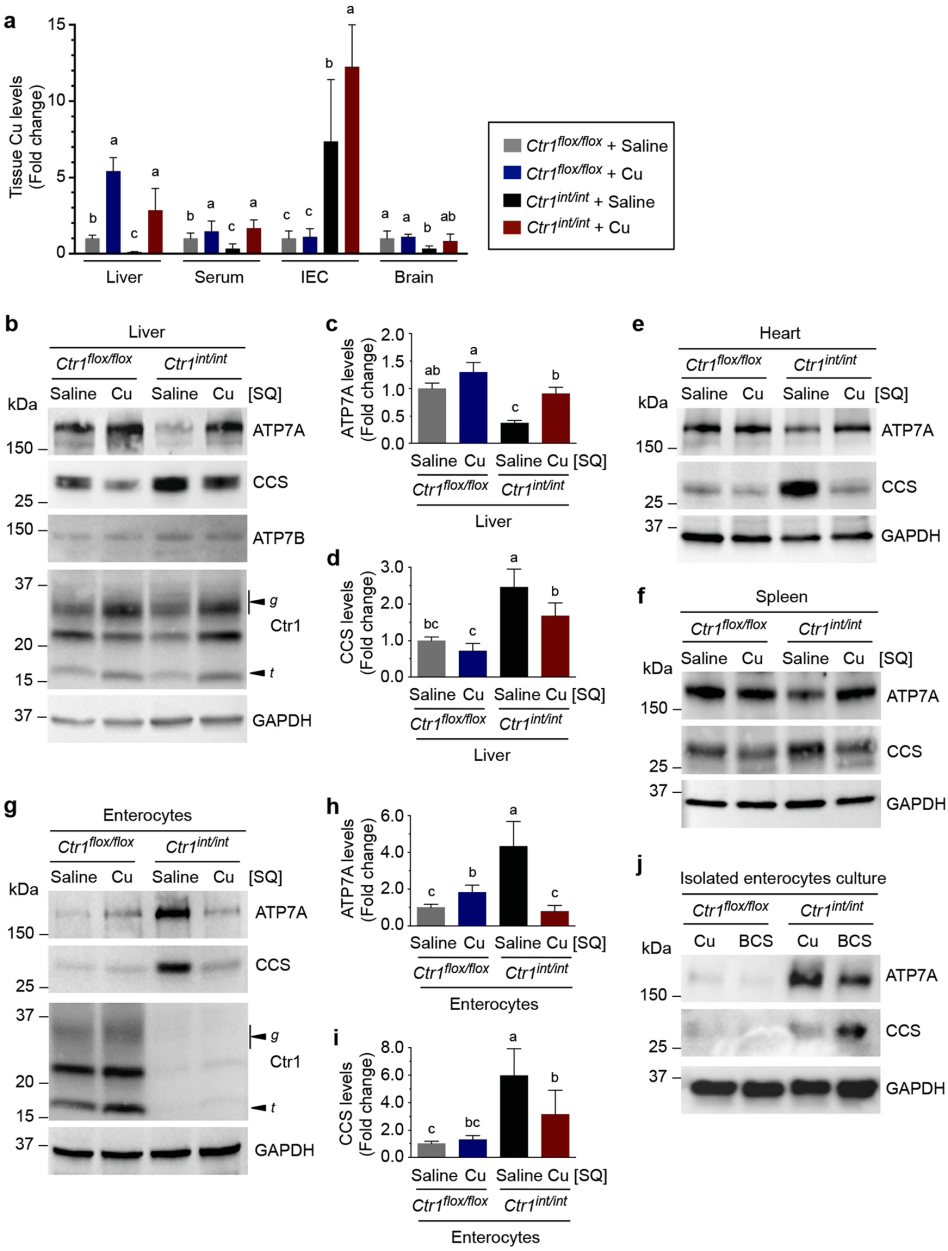
Systemic Cu status differentially regulates ATP7A protein levels in enterocytes and peripheral tissues. **(a)** Relative Cu levels in liver extracts, serum, intestinal epithelial cells (IEC), and total brain extracts of control mice (*Ctr1* ^*flox/flox*^ or *Ctr1* ^*flox/*+^) and *Ctr1* ^*int/int*^ mice SQ administered saline or 10 µg of Cu-histidine per body weight (g) at P10, normalized to those of control (*Ctr1* ^*flox/flox*^ or *Ctr1* ^*flox/*+^) mice administered saline. Data are shown as relative fold change compared with control (means ± SD) from seven to nine mice per condition (n = 2–5 mice per sex per condition), and means followed indicated with different letter superscripts are significantly different at *p* = 0.05 (two-way ANOVA, Tukey’s post hoc test). **(b**,**e**,**f** and **g**) Representative *Ctr1* ^*flox/flox*^ and *Ctr1* ^*int/int*^ mice littermates SQ administered saline or Cu-histidine at P10 and sacrificed at P12 for analysis. Protein extracts from the indicated tissues from these mice were immunoblotted with anti-ATP7A, anti-CCS, anti-ATP7B, anti-Ctr1, and anti-GAPDH antibodies. The arrowheads labeled *g* and *t* indicate the full-length glycosylated and truncated forms of Ctr1, respectively. Data shown here are representative of three to eleven independent experiments performed for each mouse tissue (liver, n = 12, 10, 12, and 10; heart, n = 6, 6, 4, and 4; spleen, n = 6, 4, 9, and 4; enterocytes, n = 22, 20, 22, and 22). **(c,d,h** and **i)** Quantification of ATP7A and CCS expression in *Ctr1* ^*flox/flox*^ and *Ctr1* ^*int/int*^ mice administered with saline or Cu. Relative protein abundances of hepatic and intestinal ATP7A (**c** and **h**) and CCS (**d** and **i**) were quantified by analyzing immunoblots of 10-22 mice tissues (liver, n = 12, 10, 12, and 10; enterocytes, n = 22, 20, 22, and 22) for each condition for statistical analysis. Error bars represent average ± SD, and means indicated with different letters are significantly different from each other at *p* = 0.05 (Two-way ANOVA, Tukey’s post hoc test). **(j)** Cu-responsiveness of isolated *Ctr1* ^*int/int*^ enterocytes cultures. Isolated enterocytes from *Ctr1* ^*flox/flox*^ and *Ctr1* ^*int/int*^ mice were cultured in medium containing 100 μM CuCl_2_ or 300 μM BCS for 2 h. Total protein extracts of cultured enterocytes were subjected to SDS-PAGE and analyzed by immunoblotting. Immunoblots shown here are representative of three independent experiments performed on a total of n = 5, 5, 7, and 7. Full-length blots are presented in Supplementary Figure 14.

The administration of Cu treatment in C57BL/6 mice did not lead to robust changes in Cu levels in the circulation (Fig. 3a) and resulted in an increase in ATP7A levels in the liver only in mice administered Cu at the concentration of 10 μg/g BW (Fig. 3g and S2a) compared to those in cell culture models (Fig. 1g) despite comparable cellular Cu levels between the *in vitro* (Fig. 1h) and *in vivo* (Fig. 3c) experimental conditions. We therefore postulated that the tissues of Cu-deficient mice may be more sensitive to Cu fluctuations in circulation than those from Cu-adequate wild-type mice. To test this hypothesis, we utilized the intestine-specific Ctr1 knockout mouse (*Ctr1* ^*int/int*^) as a Cu-deficient animal model. Ctr1 knockout in the intestine markedly reduces Cu accumulation in peripheral tissues including the liver, heart, and spleen, and results in severe Cu deficiency phenotypes at P10 ^9,33^. P10 *Ctr1* ^*int/int*^ mice were SQ administered 10 μg Cu/g BW and sacrificed after 48 h, and the steady-state levels of Cu in several peripheral tissues of *Ctr1* ^*int/int*^ and control mice (*Ctr1* ^*flox/flox*^) administered with Cu or saline were evaluated by ICP-MS. As shown in Fig. 4a, *Ctr1* ^*int/int*^ mice demonstrated significantly reduced Cu accumulation in all peripheral tissues tested. Consistent with previous data, the intestine of *Ctr1* ^*int/int*^ mice hyperaccumulated Cu in a non-bioavailable pool^9^, as supported by elevated CCS abundance representing reduced bioavailable Cu levels (Fig. 4g and i).

To explore whether fluctuations in Cu concentrations in peripheral tissues are associated with changes in ATP7A protein expression levels, we examined ATP7A levels from *Ctr1* ^*int/int*^ and *Ctr1* ^*flox/flox*^ pups administered with Cu or saline injections. ATP7A levels in the peripheral tissues (such as the livers) of *Ctr1* ^*int/int*^ mice were clearly decreased as compared to those in age-matched sibling control mice (*Ctr1* ^*flox/flox*^); however, this decrease in ATP7A levels was rescued to the levels found in control *Ctr1* ^*flox/flox*^ mice by SQ Cu administration (Fig. 4b and c). Hyper-accumulated Fe levels in the intestine and liver, likely due to a reduction of Fe efflux facilitated by the Cu-dependent ferroxidase hephaestin and ceruloplasmin^9^, were also rescued by Cu administrations in these mice (Fig. S4). The abundance of hepatic ATP7B protein was not changed by Cu injection or Cu deficiency (Fig. 4b), which is consistent with observations that elevated Cu levels in the body alters the cellular localization of ATP7B from the TGN to lysosomes without any concomitant change in ATP7B protein levels^34^.

While Cu deficiency did not affect Ctr1 abundance in the livers of *Ctr1* ^*int/int*^ mice, the glycosylated full-length form of hepatic Ctr1 was highly expressed in both Cu-treated *Ctr1* ^*int/int*^ and *Ctr1* ^*flox/flox*^ mice as compared to saline-treated *Ctr1* ^*int/int*^ and *Ctr1* ^*flox/flox*^ mice (Fig. 4b and S5), reaffirming that the liver acts as a Cu storage and sequestration organ under Cu overload conditions. Increased CCS in the livers of *Ctr1* ^*int/int*^ pups due to Cu deficiency was suppressed by Cu administration, (Fig. 4b and d), consistent with the ICP-MS analysis (Fig. 4a). ATP7A levels in several other peripheral tissues including the hearts and spleens similarly exhibited reduced ATP7A levels in *Ctr1* ^*int/int*^ mice as compared to *Ctr1* ^*flox/flox*^ mice, and these levels were restored by Cu administration (Fig. 4e,f and S6a–d). These *in vivo* findings suggest that ATP7A protein abundance in several peripheral organs is regulated in response to Cu levels in animals.

### Intestinal ATP7A protein abundance primarily responds to systemic Cu status

Since intestinal Cu absorption is the primary point at which Cu transport into circulation can be regulated, we examined the expression levels of ATP7A in intestinal enterocytes in Ctr1- and Cu-deficient mice. Interestingly, intestinal ATP7A abundance was inversely correlated with bioavailable Cu, in direct opposition to its regulation in the liver (Fig. 4b,c,g and h). While bioavailable Cu was limited in the intestinal epithelial cells, as indicated by increased levels of CCS (Fig. 4g and i), ATP7A expression was strongly elevated as compared to that in saline-treated *Ctr1* ^*flox/flox*^ mice; this elevated ATP7A was suppressed by Cu administration, leading to increases in bioavailable Cu in the intestine and other tissues as compared to that in saline-treated *Ctr1* ^*int/int*^ mice (Fig. 4g,h and i). Elevated intestinal CCS protein levels in *Ctr1* ^*int/int*^ mice were rescued by Cu-administration, indicating that bioavailable Cu was increased in the enterocytes in *Ctr1* ^*int/int*^ mice administered with Cu (Fig. 4a,g and i)^2,9,35^. This result was not anticipated, as our *Ctr1* ^−/−^ MEFs results (Fig. 1j) indicate that bioavailable Cu levels in enterocytes are severely limited, which would lead to decreased ATP7A levels. Enterocytes displayed the opposite response when compared to that of cultured cells and peripheral tissues. These intriguing observations suggest that while ATP7A acts to manage intracellular Cu homeostasis in peripheral tissues, intestinal ATP7A levels are at least partially determined by extraintestinal Cu status.

To further explore the *in vivo* regulation of ATP7A abundance under differential Cu availability, *Atp7a* mRNA levels in mouse tissues were measured by reverse transcription quantitative PCR (RT-qPCR). *Atp7a* steady-state mRNA levels did not significantly change in either the livers or the intestines of *Ctr1* ^*int/int*^ and *Ctr1* ^*flox/flox*^ mice administered Cu or saline (Fig. S7). This indicated that control of ATP7A abundance in response to Cu occurs post-transcriptionally, similar to regulation in cell cultures (Fig. 1a–c) and reminiscent of the dramatic changes in ATP7A protein, but not mRNA, in the intestine caused by cardiac-specific loss of Ctr1 in mice (*Ctr1* ^*hrt/hrt*^)^25^ (Fig. S8). Taken together, these results demonstrate that intestinal epithelial cells control ATP7A protein abundance via post-transcriptional regulation in response to peripheral tissue Cu deficiency.

The diametrically opposing effects of Cu deficiency on ATP7A expression in peripheral tissues compared to enterocytes raises the question as to what mechanism underlies communication between the intestine and Cu-deficient peripheral tissues. Isolated enterocytes from *Ctr1* ^*int/int*^ and *Ctr1* ^*flox/flox*^ mice were exposed to culture medium treated with Cu or BCS for 2 h. Immunoblot analysis of these cells revealed that elevated intestinal ATP7A in *Ctr1* ^*int/int*^ mice was decreased by BCS treatment, concomitant with an increase in CCS expression in isolated and cultured enterocytes when compared to those from the Cu-treated medium (Fig. 4j, S6e and S6f). Thus, when isolated in culture, enterocytes appear to regulate ATP7A expression in response to Cu availability in a manner that more closely resembles that of peripheral tissues and cultured cells than that found in enterocytes in their native milieu. Cu treatments of either the apical or basolateral side of polarized IEC-6 cells using a trans-well system led to elevation of ATP7A levels (Fig. S9) suggesting polarization *per se* is not sufficient to recapitulate the ATP7A phenotype shown in the intestine of Cu-injected *Ctr1* ^*int/int*^ (Fig. 4g).

To determine whether the changes in intestinal ATP7A abundance could be dependent upon other cell types found in the intestine, we examined the localization of ATP7A across the duodenum and upper jejunum of *Ctr1* ^*int/int*^ and *Ctr1* ^*flox/flox*^ mice. Multi-label confocal immunofluorescence microscopy showed that ATP7A expression was specific to enterocytes at the villus tip in the intestine (Fig. S10a–c). The majority of ATP7A was appeared in intracellular puncta in both *Ctr1* ^*int/int*^ and *Ctr1* ^*flox/flox*^ mice, with partial localization in the basolateral membranes (marked by the Na^+^/K^+^-ATPase) (Fig. S10d and e). Highly elevated levels of ATP7A were apparent in *Ctr1* ^*int/int*^ mice as compared to control mice, confirming the immunoblot data (Fig. 4g and h). Taken together, these findings indicate that highly increased intestinal ATP7A in *Ctr1* ^*int/int*^ mice primarily localizes to intracellular vesicles in the enterocytes of intestinal villi, a distribution similar to that in *Ctr1* ^*flox/flox*^ mice.

**Figure 5:**
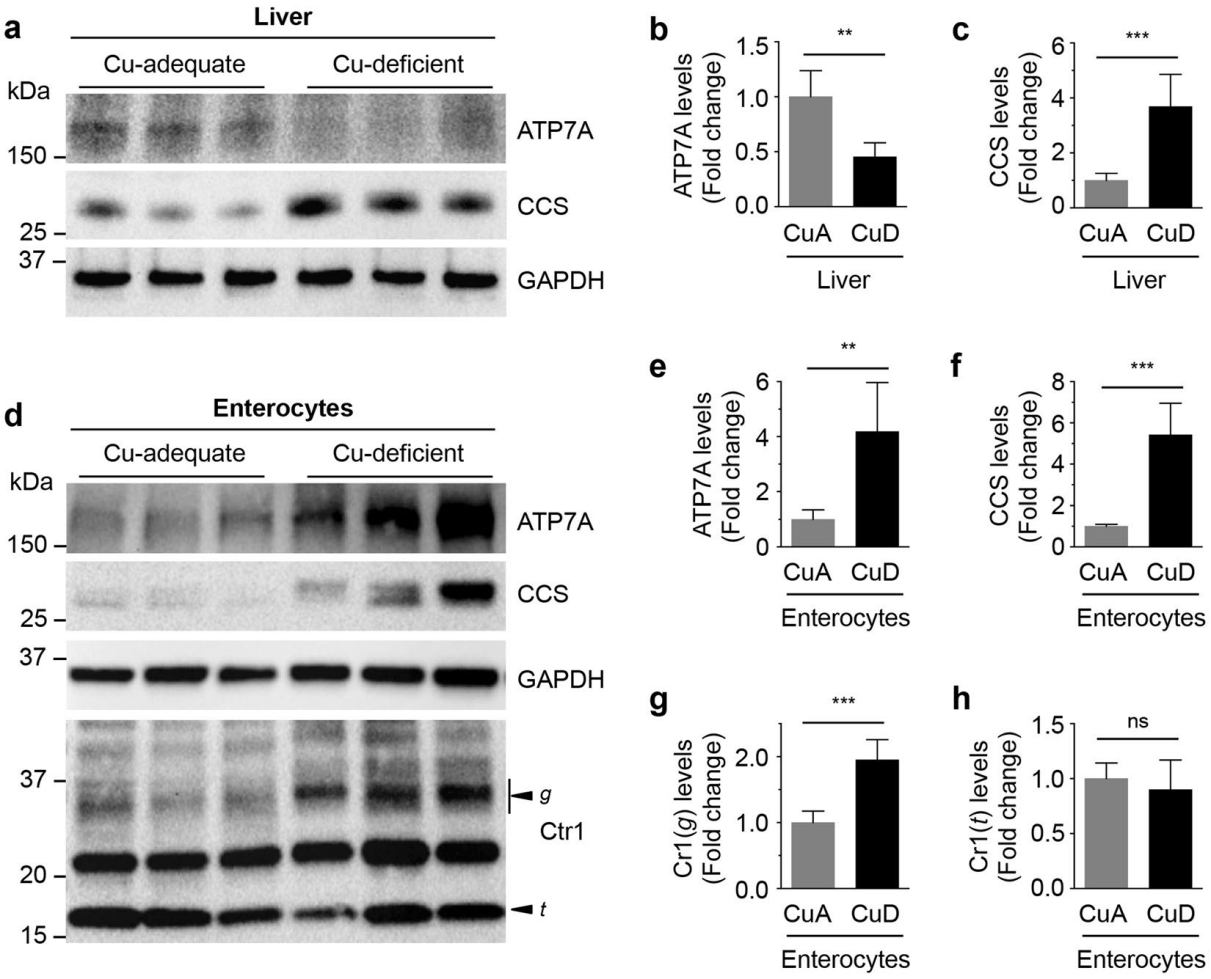
Dietary Cu-deficient mice exhibit elevated protein levels of ATP7A and Ctr1 in the intestine. (**a** and **d**) Immunoblot analysis of ATP7A and CCS in the liver (**a**) and enterocytes (**d**) of three representative mice (P15) fed a control or Cu-deficient diet. GAPDH levels were assayed as a loading control. The arrowheads labeled *g* and *t* indicate the glycosylated full-length and truncated forms of Ctr1, respectively. Data shown in here are representative of three independent experiments performed on male (n = 3 and 3) and female (n = 3 and 4) mice for Cu-adequate and Cu-deficient conditions. **(b**,**c**,**e**,**f**,**g** and **h**) Quantification of ATP7A, CCS, and Ctr1 expression in the liver and enterocytes of Cu-adequate (CuA; male, n = 3; female, n = 3) or Cu-deficient (CuD; male, n = 3; female, n = 4) mice. Error bars indicate mean ± SD. Statistics: two-tailed unpaired Student’s t-test (ns, P > 0.05, **P < 0.01, and ***P < 0.001). Full-length blots are presented in Supplementary Figure 15.

The results from the *Ctr1* ^*int/int*^ mice support the hypothesis that ATP7A expression in the enterocyte is controlled by the need to supply Cu to peripheral tissues. Alternatively, the changes to ATP7A abundance in the intestine could also be attributed to the intestinal loss of Ctr1 in *Ctr1* ^*int/int*^ mice, independent of peripheral Cu deficiency. To separate these two possibilities, we determined if intestinal ATP7A expression was regulated by Cu deficiency in wild-type mice in a similar manner to their *Ctr1* ^*int/int*^ and *Crt1* ^*hrt/hrt*^ counterparts. We performed analysis of ATP7A levels in mice fed Cu-adequate and Cu-deficient diets. Dietary Cu-deficiency in wild type mice correlated to a reduction in hepatic ATP7A expression in parallel with increased CCS expression resulting from Cu restriction (Fig. 5a–c). However, abundance of the intestinal ATP7A Cu efflux pump was significantly enhanced, even while enterocytes demonstrated severe Cu-deficiency, as indicated by elevated levels of CCS (Fig. 5d–f), similar to the observations from *Ctr1* ^*int/int*^ mice, demonstrating that increased ATP7A abundance in the intestine is dependent on peripheral Cu deficiency and not on intestinal loss of Ctr1. Moreover, enterocytes expressed higher amounts of the glycosylated full-length form of Ctr1 in the intestinal epithelial cells of Cu-deprived mice (Fig. 5d,g and h) suggesting the mechanism for Cu-deficiency induced stabilization of Ctr1 is conserved in the enterocytes as observed in cell-culture models. Together, these findings suggest an intestinal regulatory mechanism for dietary Cu absorption via increased expression of the Ctr1 Cu importer and ATP7A Cu exporter in wild type mice, and indicate that intestinal ATP7A regulation is distinct from that in the liver and other peripheral organs.

## Discussion

The unique physiological functions and specific cell types found in various mammalian organ systems result in differential demands for Cu. The role of the Cu exporter ATP7A in cellular Cu delivery to secretory pathways and into peripheral circulation in organisms has been well established via its Cu-responsive trafficking^7,16,36–38^. However, the mechanisms by which ATP7A abundance in distinct tissues responds to changes in organismal Cu remain poorly understood^2,13,25^. Here we present data that suggest ATP7A abundance is an important regulatory point for Cu homeostasis in peripheral tissues including the heart and spleen; in mice, excess Cu results in increased ATP7A protein levels in liver tissue, consistent with a need to prevent over-accumulation of this metal through enhanced ATP7A-driven Cu export. Our data demonstrate that in response to potentially detrimental Cu concentrations, cell systems not only relocate ATP7A to post-Golgi vesicles and the plasma membrane^16^, but also increase the protein abundance of ATP7A in order to enhance export of Cu from cells and restore intracellular Cu homeostasis (Fig. 1 and 2)^21^. Conversely, under Cu deficiency, our results suggest that ATP7A protein abundance is reduced, at least in part, by the proteasome system (Fig. 2b), consistent with a reduced need to export Cu from the cell. It is worth noting that Cu status regulates the multicopper oxidase hephaestin via a proteasome-mediated pathway^39^, and that XIAP targets CCS for ubiquitination through its E3 ubiquitin ligase activity, resulting in the degradation of CCS under adequate levels of Cu^40^. It is possible that a similarly-regulated E3 ubiquitin ligase binds to ATP7A and targets it for degradation during Cu deficiency.

While it is known that the liver is a major Cu storage organ in mammals, little attention has been paid to the delineation of the biochemical mechanisms by which mammals regulate the detoxification, storage, and mobilization of Cu^41^. We observed that Cu accumulation is drastically increased in the liver as compared to other peripheral tissues in mice administered SQ Cu (Fig. 3c and 4a), supporting the concept of the liver as the principle Cu storage organ. Ctr1 is known to be internalized and degraded in response to high Cu levels in cultured cells^32^, which we also observed in this study (Fig. 1i). Unexpectedly, the liver demonstrated an increase in the mature glycosylated form of Ctr1 in response to Cu administration when compared to levels in mice administered saline (Fig. 3g and 4b). This finding suggests that in contrast to other tissues, liver Ctr1 expression is elevated under high Cu conditions; this is possibly to facilitate removal of Cu from the circulation and prevent build up in other Cu-susceptible peripheral tissues. As such, it is plausible that the responsiveness of Ctr1 in the liver *in vivo* is different from that in cell culture because organismal Cu homeostasis is coordinated by multiple layers of regulation, and likely influenced by one or more additional Cu-dependent circulatory factor(s) rather than changes in Cu concentrations alone. The increased hepatic ATP7A protein may function to export Cu in response to Cu overload when Cu exceeds the capacity of the liver as suggested in cell culture studies (Fig. 1). An alternate possibility would be that ATP7A is upregulated to maintain uninterrupted Cu supply to secretory pathways for Cu dependent enzymes, while ATP7B traffics to the apical membrane to excrete surplus Cu in the liver. The mechanisms underlying the contrasting responses of Ctr1 in liver versus other tissues remain to be determined.

Unexpected observations from our analyses of Cu-deficient mice include the elevated protein levels of ATP7A and glycosylated full-length form of Ctr1 in enterocytes associated with limited levels of bioavailable intracellular Cu, while under these same conditions, peripheral tissues showed significantly diminished ATP7A expression. Additionally, while Cu accumulation was significantly increased in the enterocytes of *Ctr1* ^*int/int*^ mice, SQ Cu administration into these mutant mice suppressed intestinal ATP7A expression and increased Ctr1 truncation, which would limit dietary Cu import by the reduced Cu transport activity (Fig. 4a,g,h and S5)^42,43^. A previous study revealed that cardiac-specific Ctr1 ablation in mice (*Ctr1* ^*hrt/hrt*^) results in a dramatic upregulation of ATP7A expression in both the intestine and liver and a concomitant elevation of serum Cu levels^25^. Our results raise the possibility that modulation of ATP7A abundance in the intestine, not the liver, is the principal means by which Cu supply to circulation is regulated depending upon Cu demands from peripheral tissues. Generation and analysis of liver-specific ATP7A knockout mice could delineate the role of ATP7A and physiological significance of its abundance regulation in the liver. Notably, changes in Cu levels did not significantly alter the subcellular distribution of ATP7A in enterocytes (Fig. S10). This strengthens the possibility that *in vivo*, the intestine increases ATP7A abundance in intracellular vesicles to efflux Cu via exocytosis through basolateral membrane into circulation as proposed by others^36^.

Intestinal epithelial cells in *Ctr1* ^*int/int*^ mice administered with SQ Cu exhibited profound Cu accumulation in parallel with enhanced levels of bioavailable Cu as indicated by decreased CCS, and a rescued Fe hyper-accumulation phenotype when compared with control (saline-administered) *Ctr1* ^*int/int*^ mice (Fig. 4a,g,i and S4). Given that Ctr1 localizes to the apical membrane in enterocytes^33^, and intestinal Ctr1 is poorly expressed in *Ctr1* ^*int/int*^ mice^9^, these observations suggest the existence of alternative Cu uptake machinery at the basolateral membrane of intestinal epithelial cells. Such a mechanism may have its origins *in utero* where Cu delivery to enterocytes might occur via Ctr1-independent serosal-to-mucosal Cu transport. A possible pathway in this process might be an anion exchanger-dependent Cu import system^44^, which remains to be identified.

Studies of cultures of isolated enterocytes from *Ctr1* ^*int/int*^ mice presented here show that elevated ATP7A expression in the enterocytes of Cu-deficient mice is suppressed in culture with BCS-treated medium (Fig. 4j), whereas identical enterocytes showed stronger ATP7A expression concomitant with reduced serum Cu levels as compared to *Ctr1* ^*flox/flox*^ mice (Fig. 4g). These observations underscore the importance of the intestine as a regulator of Cu entry to the body, which must respond not only to intrinsic Cu needs, but those of peripheral tissues via the basolateral side of epithelial cells that is thought to be in direct communication with circulating effectors. As intestinal ATP7A levels in both *Ctr1* ^*int/int*^ (low serum Cu)^9^, and *Ctr1* ^*hrt/hrt*^ (high serum Cu)^25^ mice are elevated, the data suggest that serum Cu itself is not a direct intestinal ATP7A inducer. Instead, we speculate that a circulating factor likely regulates intestinal levels of ATP7A. Clearly, more extensive studies are required to identify such a molecule and its mode of action. Such studies will greatly expand our understanding of the regulation of systemic Cu homeostasis and human diseases caused by Cu dysregulation.

## Methods

### Antibodies

The anti-ATP7A antibody (generous gift from Drs. Stephen G. Kaler, National Institutes of Health (NIH), Bethesda, MD and Michael J. Petris, University of Missouri, Columbia, MO), anti-ATP7B antibody (generous gift from Dr. Svetlana Lutsenko, Johns Hopkins University, Baltimore, MD), anti-Ctr1 antibody^9^, anti-β-tubulin antibody (Cell Signaling Technology), anti-β-actin antibody (Sigma), and anti-Na^+^/K^+^-ATPase (Thermo Scientific) antibody were each used at a 1:1,000 dilution. The anti-CCS antibody (Santa Cruz Biotechnology) and anti-GAPDH antibody (Sigma) were used at a 1:400 dilution and 1:10,000 dilution, respectively. Horseradish peroxidase-conjugated anti-rabbit or anti-mouse IgG (Rockland Immunochemicals) were used as the secondary antibody for immunoblotting at a 1:5,000 dilution. Immunoblots were detected using SuperSignal West Pico or Femto Chemiluminescent Substrate reagents (Thermo Fisher Scientific) using a chemidocumentation imaging system (Bio-Rad). The anti-Na^+^/K^+^-ATPase antibody was purchased from the Developmental Studies Hybridoma Bank (DSHB) for immunofluorescence experiments. Alexa Fluor 488 anti-rabbit IgG, Alexa Fluor 568 anti-mouse IgG, Alexa Fluor 647 WGA-conjugate, and ProLong Gold Antifade mounting reagent with DAPI were obtained from Life Technologies.

### Cell Culture

Rat intestinal epithelial cells (IEC-6) and HUVEC cells were obtained from the American Type Culture Collection (ATCC) and cultured according to the distributor’s recommendations. Wild-type (*Ctr1* ^+/+^) and *Ctr1* ^*−/−*^ MEFs were cultured in Dulbecco’s Modified Eagle Medium (DMEM; Lonza) supplemented with 10% (v/v) heat-inactivated fetal bovine serum (FBS; Atlanta Biologicals), 1 × MEM non-essential amino acids (Lonza), 50 µg/mL uridine, 100 U/mL penicillin/streptomycin (Lonza), and 55 µM 2-mercaptoethanol. All cells were cultured under 5% CO_2_ at 37 °C. Cells at ~60% confluence were collected and washed three times with ice-cold PBS (pH 7.4). CuCl_2_ (Alfa Aesar) and bathocuproinedisulfonic acid Cu chelator (BCS; Acros) were used for Cu level-responsive ATP7A expression change experiments with various cell lines. For proteasomal degradation inhibition experiments, IEC-6 cells were treated with 50 μg/mL cycloheximide (CHX; Sigma), 20 μM MG-132 (Sigma), and 100 nM bortezomib (ApexBio). Cell pellets were suspended in ice-cold cell lysis buffer (PBS pH 7.4, 1% Triton X-100, 0.1% sodium dodecyl sulfate (SDS), 1 mM EDTA) containing Halt protease inhibitor cocktail (Thermo Scientific), briefly vortexed, and incubated for 1 h. Cell suspensions were centrifuged at 16,000 x*g* at 4 °C for 15 min to remove insoluble material, and supernatants were collected. Protein concentrations were measured using the BCA Protein Assay Kit (Thermo Scientific). The indicated amount of protein extract was fractionated by SDS-gel electrophoresis on 4-20% gradient gels (Bio-Rad) and immunoblotted.

IEC-6 cells were grown on 24-mm polyester membrane transwells with 0.4-μm pores (Corning) until confluent. When cultures reach to 90% confluence, the growth medium was exchanged every day to produce a polarized cell monolayer. Formation of tight junctions was monitored by the measurement of trans epithelial electrical resistance (TEER) using an EVOM2 meter and STX2 electrodes (World Precision Instruments). Cells were judged to be ready for Cu uptake experiments when the TEER value was ~400 Ω × cm^2^. After incubating cells with 200 μM BCS for 24 h, both compartments were filled with fresh culture medium containing BCS, CuCl_2_, or 10 mM EDTA for 12 h. Cells were rinsed with ice-cold PBS (pH 7.4), scraped in the same ice-cold lysis buffer to harvest, and analyzed by immunoblotting.

Primary liver cells were isolated from mice at P12 according to the procedure previously described in detail^45^. Briefly, an incision was made on the right atrium with scissors to ensure a route for overflow, then perfusion was performed with calcium-free Hanks’ balanced salt solution (HBSS; Lonza) supplemented with 0.5 mM EDTA (Sigma) at 37 °C at a rate of 3.5 mL/min, followed by HBSS supplemented with 0.3 mg/mL collagenase (Sigma) and 5 mM CaCl_2_ under the same conditions at a rate of 5 mL/min. The total-collected liver cell suspension was then filtered through a 100-µm nylon mesh and centrifuged at 50 × *g* for 5 min. Next, the pellet was resuspended and incubated in DMEM with 10% FBS, 0.1 µM insulin (Sigma), and 100 U/mL penicillin/streptomycin. After 3 h of incubation at 37 °C in an atmosphere of 5% CO_2_, the medium was changed to M199 supplemented with 0.1 µM dexamethasone, 0.1 µM 3,3′,5-triiodo-L-thyronine, 0.1 µM insulin, and 100 U/mL penicillin/streptomycin and incubated for further analysis.

### Animal and Tissue Preparations

For the Cu administration experiment, pups at P7 (C57BL/6J, Jackson Laboratory) were SQ administered 50 μL of saline containing 10 μg CuCl_2_ with 19.12 μg L-histidine/g mouse BW or 50 μL of saline alone for 3 consecutive days and sacrificed at P10. The intestinal Ctr1 knockout mice (*Ctr1* ^*int/int*^) and control mice (*Ctr1* ^*flox/flox*^ and *Ctr1* ^*flox/*+^) were generated as described previously^9^. *Ctr1* ^*flox/flox*^ and *Ctr1* ^*int/int*^ mice pups were administered the same amount of Cu-histidine or saline alone given subcutaneously at P10 and dissected two days later (P12). To generate dietary Cu-deficient mice, dietary treatment of dams began on embryonic day 16 or 17, as previously described^8^. Dams from wild type mice were fed Cu-deficient or Cu-adequate diets, consisting of a Cu-deficient purified diet (TD.80388, Envigo) and either sterilized distilled low-Cu drinking water or Cu-supplemented drinking water (20 μg CuCl_2_/mL), respectively. Pups were sacrificed at P15 due to clear evidence of severe Cu deficiency. All procedures involving animals were carried out in accordance with the National Institutes of Health Guide for the Care and Use of Laboratory Animals and have been approved by the Institutional Animal Care and Use Committee at the University of Maryland, College Park (protocol number R-15-14). Tissues were dissected following washing with ice-cold PBS, frozen in liquid nitrogen, and stored at −80 °C until use. For the isolation of intestinal epithelial cells, the small intestine was opened along the long axis, washed in ice-cold PBS, and soaked in PBS containing 1.5 mM EDTA and protease inhibitors at 4 °C for 30 min with gentle agitation^46^. After incubation, the mesenchymal layer was removed, and intestinal epithelial cells were washed three times with ice-cold PBS with protease inhibitors and recovered by centrifugation at 2000 × *g* for 3 min.

Dissected tissues or purified intestinal epithelial cells were homogenized in ice-cold cell lysis buffer (PBS pH 7.4, 1% Triton X-100, 0.1% SDS, 1 mM EDTA) containing Halt protease inhibitor cocktail (Thermo Scientific) in 1.5 mL centrifuge tubes by pellet mixer (VWR) on ice. Homogenates were incubated on ice for 1 h followed by centrifugation at 16,000 × *g* for 15 min at 4 °C. The supernatants were used as total extracts for immunoblotting. Protein concentrations were measured using the BCA Protein Assay Kit (Thermo Scientific) with bovine serum albumin as a standard, and equal amounts of protein (100 μg/lane) were fractionated on a 4–20% gradient gel (Bio-Rad).

### Confocal Immunofluorescence Microscopy

For immunofluorescence analysis, intestines were washed with PBS and fixed in 4% (w/v) paraformaldehyde/PBS immediately following dissection. A 2-cm section of upper jejunum, positioned 1 cm from the stomach, was dissected and fixed with the same fixative solution overnight at 4 °C with gentle shaking. Fixed tissue samples were processed and embedded into a paraffin block and sectioned at a thickness of 5 μm for immunofluorescence staining. The deparaffinized sections were heated at a sub-boiling temperature in 1 mM EDTA buffer (pH 8.0) for 15 min to expose the antigen. Samples were blocked with 2% (w/v) BSA and 0.2% Triton X-100 in PBS for 1 h and incubated with primary antibody (as described in the figure legends) for 1 h at room temperature. After washing with 0.2% BSA and 0.02% Triton X-100 in PBS, sections were incubated with Alexa Fluor 488 anti-rabbit IgG or Alexa Fluor 568 anti-mouse IgG (1:250 dilution in 1% (w/v) BSA in PBS) for 1 h at room temperature and washed with PBS. Then, the procedure of blocking and incubation with primary and secondary antibody was repeated as described above. Following incubation with Alexa Fluor 647 WGA-conjugate for 1 h at room temperature followed by washing, sections were mounted with Gold antifade reagent with DAPI, incubated overnight at 4 °C, and visualized with a Leica SP5 confocal microscope.

### Reverse Transcription Quantitative PCR (RT-qPCR) Analysis

Total RNA was isolated from intestinal epithelial cells or liver tissues by TRIzol reagent (Invitrogen). After eliminating genomic DNA using DNA-free DNA Removal Kit (Ambion), first strand cDNA was synthesized using SuperScript VILO Master Mix (Invitrogen). RT-qPCR was performed on an Agilent Mx3005P QPCR System Thermocycler (Agilent Genomics) using SYBR Green qPCR Master Mixes (Applied Biosystems). Levels of *Atp7a, Ctr1, Mt1*, and *Mt2* mRNA were compared to an internal *Gapdh* control in mouse tissues, and rat *Atp7a* transcript levels were compared to *18s rRNA* control in IEC-6 cells..The fold change was determined using the 2^(−ΔΔCt)^ method^47^. The oligonucleotide primer sequences used for PCR were: *mAtp7a*, 5′-ATGGAGCCAAGTGTGGATG-3′ and 5′-CCAAGGCAGAGTCAGTGGAG-3′; *mGapdh*, 5′-ATGGTGAAGGTCGGTGTGAA-3′ and 5′-AGTGGAGTCATACTGGAACA-3′; *mMT1*, 5′-CACTTGCACCAGCTCCTG-3′ and 5′-GAAGACGCTGGGTTGGTC-3′; *mMT2*, 5′-CAAACCGATCTCTCGTCGAT-3′ and 5′- AGGAGCAGCAGCTTTTCTTG-3′; *mCtr1*, 5′-GGGCTTACCCTGTGAAGACTTTT-3′ and 5′-AATGTTGTCGTCCGTGTGGT-3′; *rat Atp7a*, 5′-TGAACAGTCATCACCTTCATCGTC-3′ and 5′-TGCATCTTGTTGGACTCCTGAAAG-3′; *rat 18s rRNA*, 5′-GCAATTATTCCCCATGAACG-3′ and 5′-GGCCTCACTAAACCATCCAA-3′.

### Tissue Metal Measurements

Cu and Fe concentrations were measured from nitric acid-digested cells or tissues by inductively coupled plasma mass spectrometry (ICP-MS) as described^48^. Tissues were collected into acid-washed 1.5-mL microcentrifuge tubes and weighed, and the harvested cultured cells were washed with PBS three times and weighed. The values were normalized by wet weight (g) of cells or mouse tissues. More extensive details can be found in previous reports^48^.

### Statistics

Statistical significance was determined using a one-way or two-way ANOVA followed by Tukey’s post hoc test or two-tailed unpaired Student’s t-test in GraphPad Prism, Version 6 (GraphPad Software). All data are presented as mean ± SD, and *P*-values less than 0.05 were considered statistically significant.

## Electronic supplementary material

Supplementary Information
