# Supplementary material for: Organ-specific regulation of ATP7A abundance is coordinated with systemic copper homeostasis
Source: Sci Rep. 2017 Sep 20;7:12001. doi: 10.1038/s41598-017-11961-z (PMC5607234; doi:10.1038/s41598-017-11961-z)
Supplement: Supplementary file 1 — Supplementary Information [file 41598_2017_11961_MOESM1_ESM.docx]

**Supplementary Information**

**Organ-specific regulation of ATP7A abundance is coordinated with systemic copper homeostasis**

**Haarin Chun^1^, Tracy Catterton^1^, Heejeong Kim^3^, Jaekwon Lee^3^ and Byung-Eun Kim^1,2,*^**

^1^Department of Animal and Avian Sciences, University of Maryland, College Park, MD 20742, USA

^2^Biological Sciences Graduate Program, University of Maryland, College Park, MD 20742, USA

^3^Department of Biochemistry and Redox Biology Center, University of Nebraska, Lincoln, NE 68516, USA

^*^[bekim@umd.edu](mailto:bekim@umd.edu)

**TABLE OF CONTENTS**

**Supplementary Figure 1.** Evaluation of anti-ATP7A antibody specificity and regulation of recombinant-tagged ATP7A abundance by Cu.

**Supplementary Figure 2**. Quantification of ATP7A and Ctr1 expression in the liver from WT mice SQ administered saline or Cu.

**Supplementary Figure 3**. Ctr1 protein levels in the heart, spleen, brain, and liver from WT mice SQ administered saline or Cu.

**Supplementary Figure 4**. Relative Fe levels in control mice and *Ctr1^int/int^* mice SQ administered saline or Cu.

**Supplementary Figure 5**. Quantification of Ctr1 expression in the liver and enterocytes from *Ctr1^flox/flox^* and *Ctr1^int/int^* mice administered with saline or Cu.

**Supplementary Figure 6.** Quantification of ATP7A and CCS expression in the heart and spleen from *Ctr1^flox/flox^* and *Ctr1^int/int^* mice administered with saline or Cu and isolated enterocytes culture from *Ctr1^flox/flox^* and *Ctr1^int/int^* mice.

**Supplementary Figure 7.** Reverse transcription quantitative PCR (RT-qPCR) analysis of *Atp7a* mRNA levels in the liver and intestine of *Ctr1^flox/flox^* and *Ctr1^int/int^* mice SQ administered saline or Cu.

**Supplementary Figure 8.** Protein and mRNA levels of intestinal ATP7A in cardiac-specific Ctr1 knock-out mice. **Supplementary Figure 9.** ATP7A protein levels in polarized IEC-6 cells treated with Cu or BCS.

**Supplementary Figure 10.** Confocal microscopy analysis of endogenous ATP7A in the jejunum from *Ctr1^flox/flox^* and *Ctr1^int/int^* mice.

**Supplementary Figure 11.** Full-length immunoblot images in Figure 1.

**Supplementary Figure 12.** Full-length immunoblot images in Figure 2.

**Supplementary Figure 13.** Full-length immunoblot images in Figure 3.

**Supplementary Figure 14.** Full-length immunoblot images in Figure 4.

**Supplementary Figure 15.** Full-length immunoblot images in Figure 5.

**Supplementary Figure 16.** Full-length immunoblot images in Supplementary Figure 3.

**Supplementary Figure 17.** Full-length immunoblot images in Supplementary Figure 9.

**
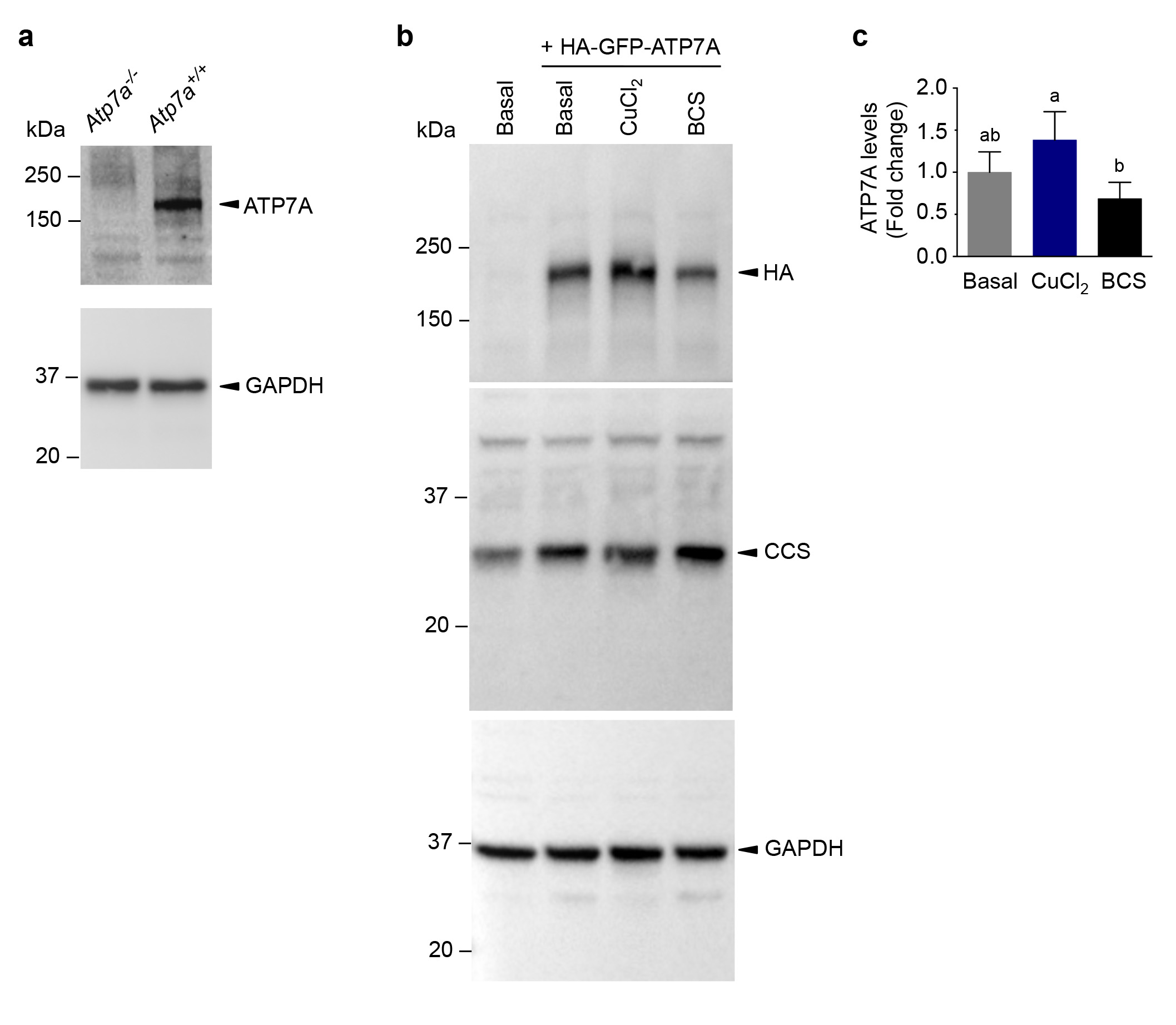
**

**Supplementary Figure 1. Evaluation of anti-ATP7A antibody specificity and regulation of recombinant-tagged ATP7A abundance by Cu.** (a) Immunoblotting with anti-ATP7A antibody. Total protein extracts from mouse embryonic fibroblasts (MEFs) of WT (*Atp7a^+/+^*) and ATP7A knock-out (*Atp7a^−/−^*) mice^1^ were assayed. The upper panel shows immunoblot results with the anti-ATP7A antibody. The lower panel shows immunoblot results with anti-GAPDH antibody as a loading control. (b and c) *Atp7a^−/−^* MEFs stably transfected with an empty vector or a CMV-driven plasmid expressing a HA-GFP-ATP7A were exposed to basal medium, basal medium containing 100 µM CuCl_2_, or 300 µM BCS for 12 h. Whole cell lysates were processed for immunoblotting analysis using antibodies as indicated. Representative immunoblots of three and five independent experiments are shown in (a) and (b and c), respectively. Means indicated with different letter superscripts are significantly different at *p*=0.05 (one-way ANOVA, Tukey’s post hoc test).

**
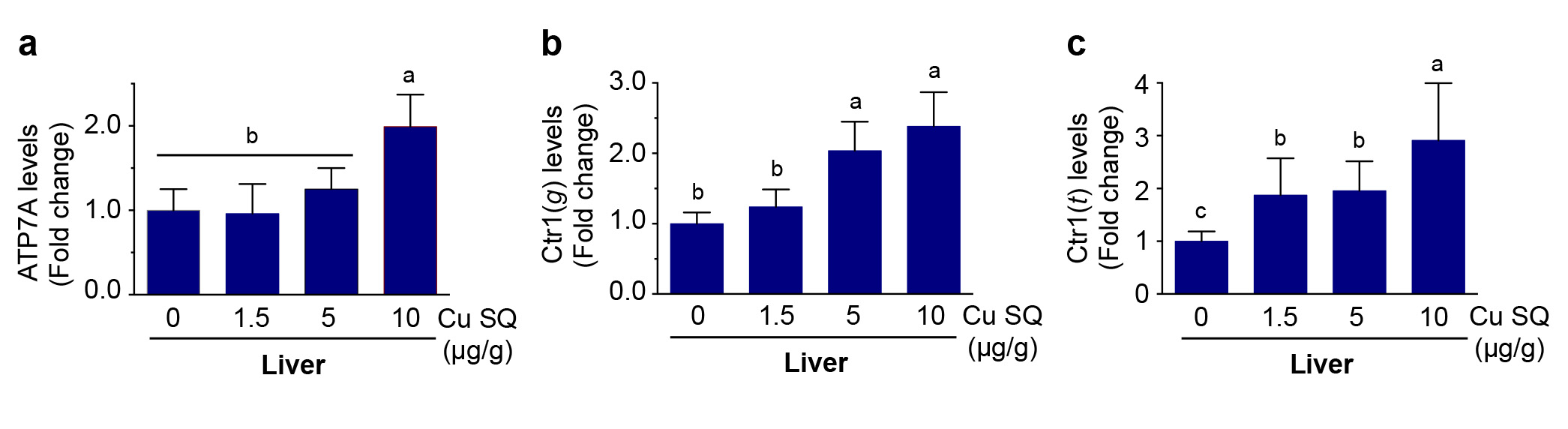
**

**Supplementary Figure 2. Quantification of ATP7A and Ctr1 expression in the liver from WT mice SQ administered saline or Cu.** Relative protein abundances of ATP7A (a) and Ctr1 (b and c) were quantified by analyzing immunoblots of liver extracts from mice (male, n = 5, 4, 4, and 6; female, n = 6, 4, 4, and 5), which were SQ administered with 0, 1.5, 5, and 10 µg CuCl_2_–histidine per body weight (g) for statistical analysis. Ctr1 (*g*) and Ctr1 (*t*) indicate glycosylated full-length and truncated form of Ctr1, respectively. Error bars represent average ± SD, and means indicated with different letters are significantly different from each other at *p*=0.05 (Two-way ANOVA, Tukey’s post hoc test).

**
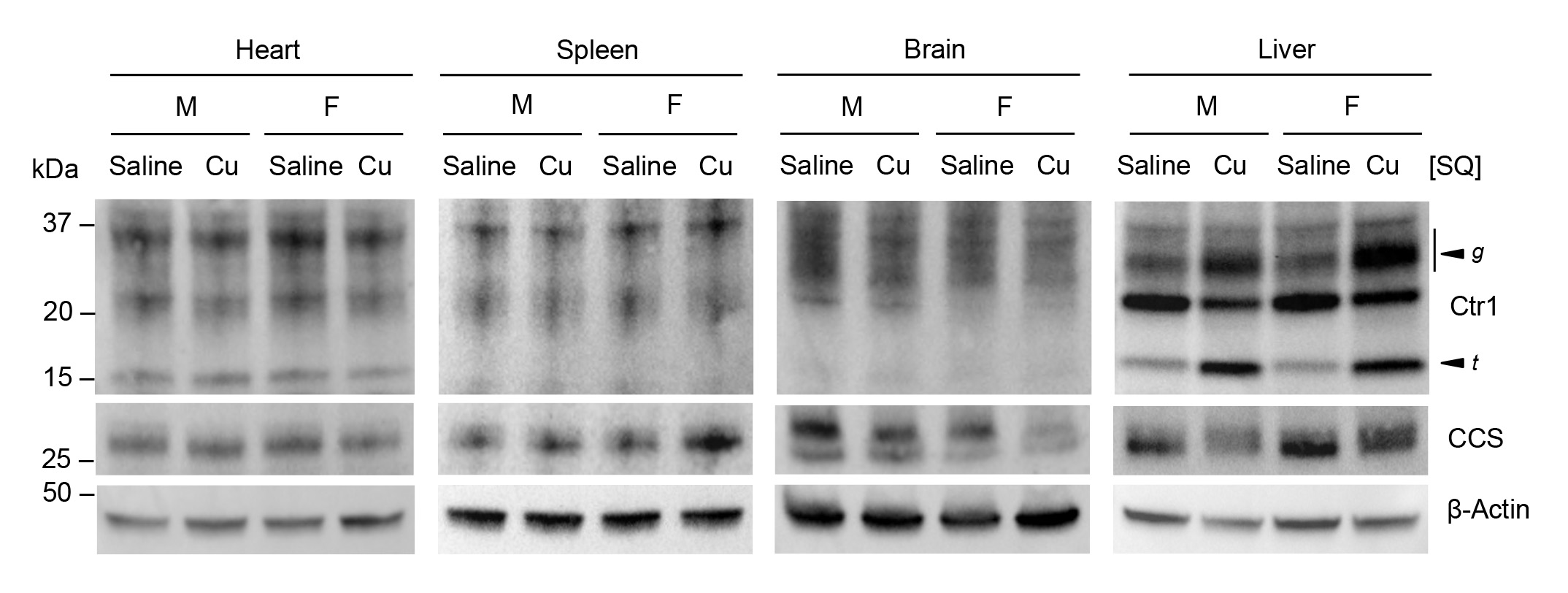
**

**Supplementary Figure 3**. **Ctr1 protein levels in the heart, spleen, brain, and liver from WT mice SQ administered saline or Cu.** Immunoblot analysis of Ctr1, CCS, and β-actin in the heart, spleen, brain, and liver extracts from two representative mice administered with saline or 10 µg of Cu-histidine per body weight (g) for three consecutive days beginning at P7. The arrowheads indicate the full-length glycosylated (*g*) and truncated form (*t*) of Ctr1, respectively. β-actin levels were assayed as a loading control. Data shown here are representative of three to four independent experiments performed for each tissue of male (M) (liver, n = 5 and 6; heart, n = 5 and 6; brain, n = 2 and 2; spleen, n = 3 and 4) and female (F) mice (liver, n = 6 and 5; heart, n = 6 and 5; brain, n = 2 and 2; spleen, n = 3 and 2), which were SQ administered saline or Cu. Full-length blots are presented in Supplementary Figure 16.

**
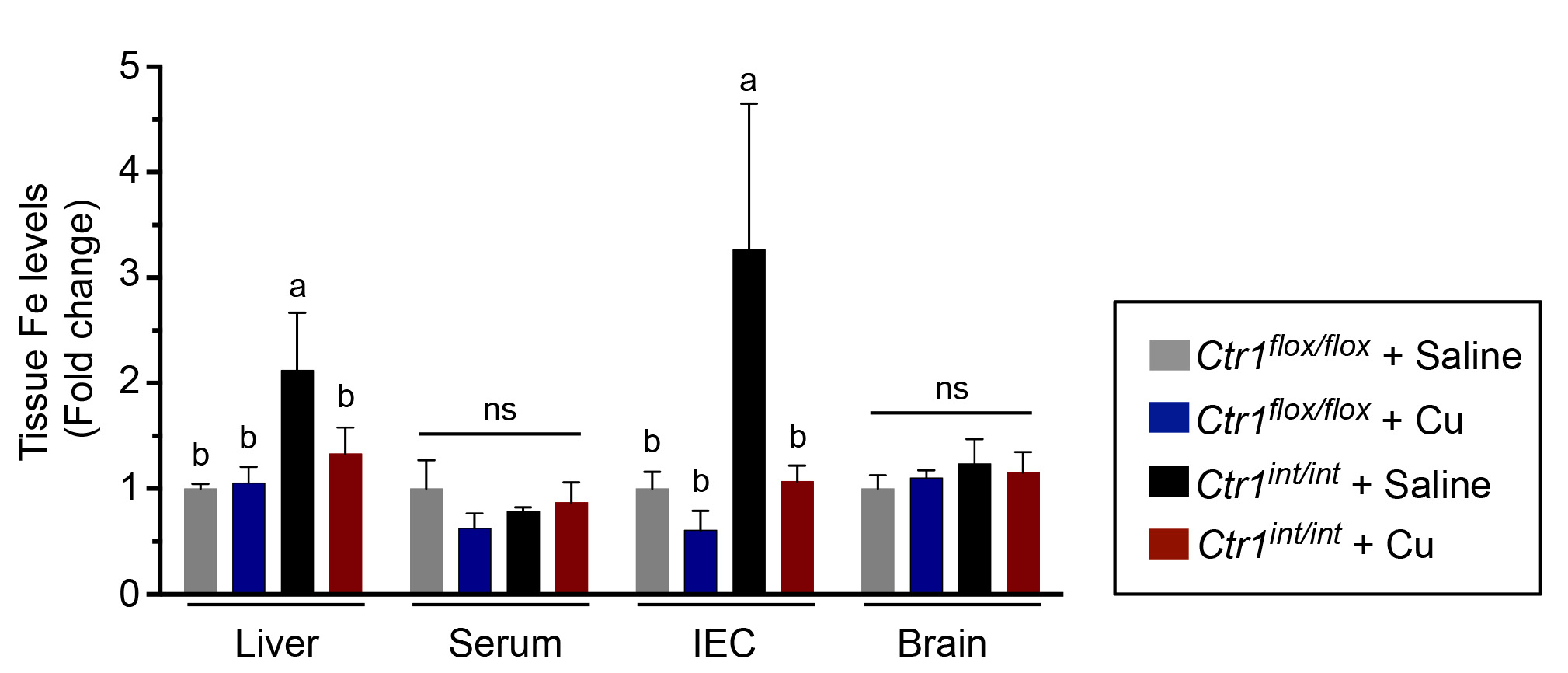
**

**Supplementary Figure 4. Relative Fe levels in control mice and *Ctr1^int/int^* mice SQ administered saline or Cu.** Relative Fe levels in liver extracts, serum, intestinal epithelial cells (IEC), and total brain extracts of control mice (*Ctr1^flox/flox^* or *Ctr1^flox/+^*) and *Ctr1^int/int^* mice SQ administered saline or Cu-histidine at P10, normalized to those of control (*Ctr1^flox/flox^* or *Ctr1^flox/+^*) mice administered saline. Data are shown as relative fold change compared with control (means ± SD) from seven to nine mice per condition (n = 2-5 mice per sex per condition), and means indicated with different letter superscripts are significantly different at *p*=0.05 (two-way ANOVA, Tukey’s post hoc test).

**
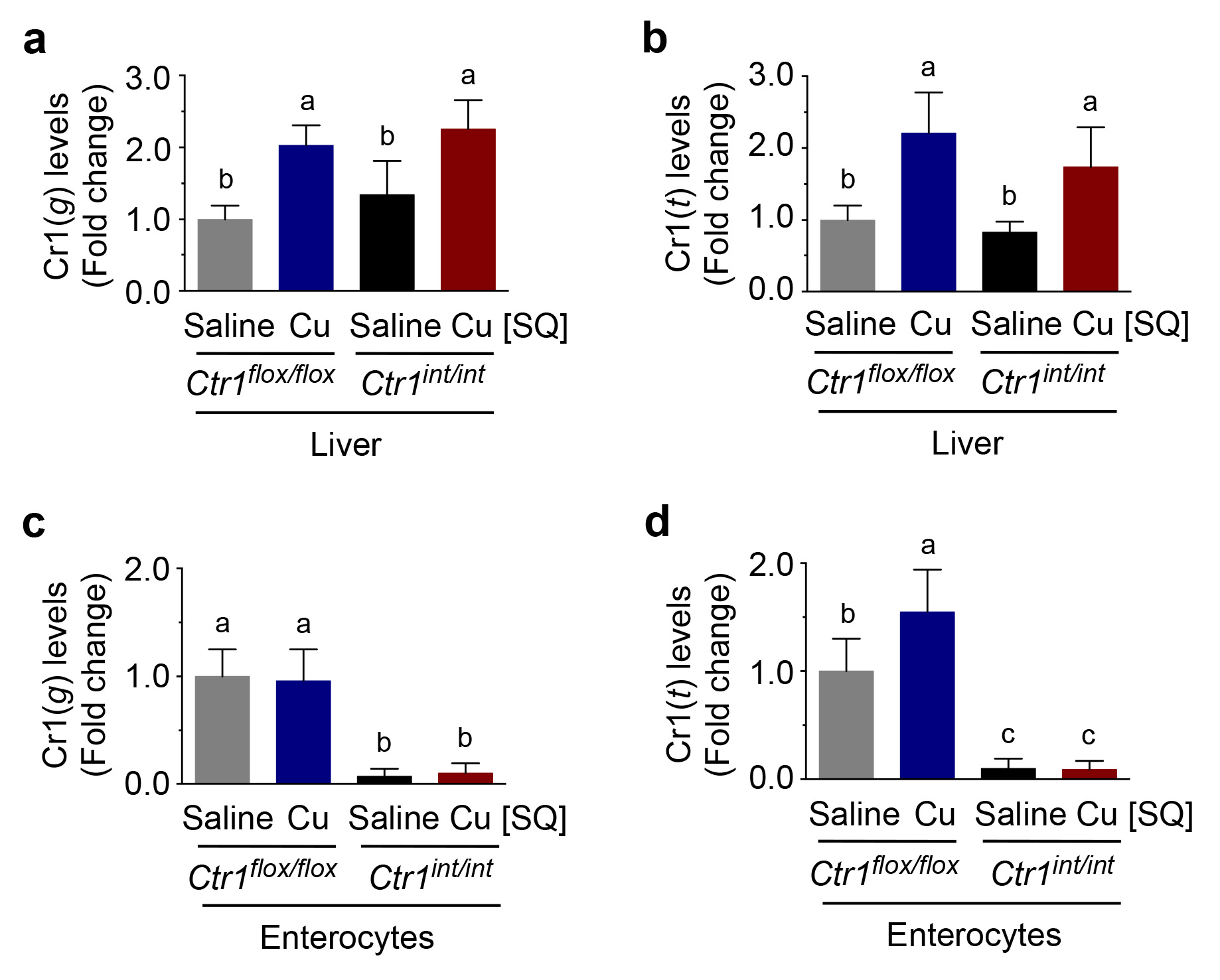
**

**Supplementary Figure 5. Quantification of Ctr1 expression in the liver and enterocytes from *Ctr1^flox/flox^* and *Ctr1^int/int^* mice administered with saline or Cu.** Relative protein abundances of Ctr1 were quantified by analyzing immunoblots of each tissue from mice (liver, n = 7, 7, 7, and 7; enterocytes, n = 6, 6, 6, and 6) for each condition for statistical analysis. Ctr1 (*g*) and Ctr1 (*t*) indicate glycosylated full-length and truncated form of Ctr1, respectively. Error bars represent average ± SD, and means indicated with different letters are significantly different from each other at *p*=0.05 (Two-way ANOVA, Tukey’s post hoc test).

**
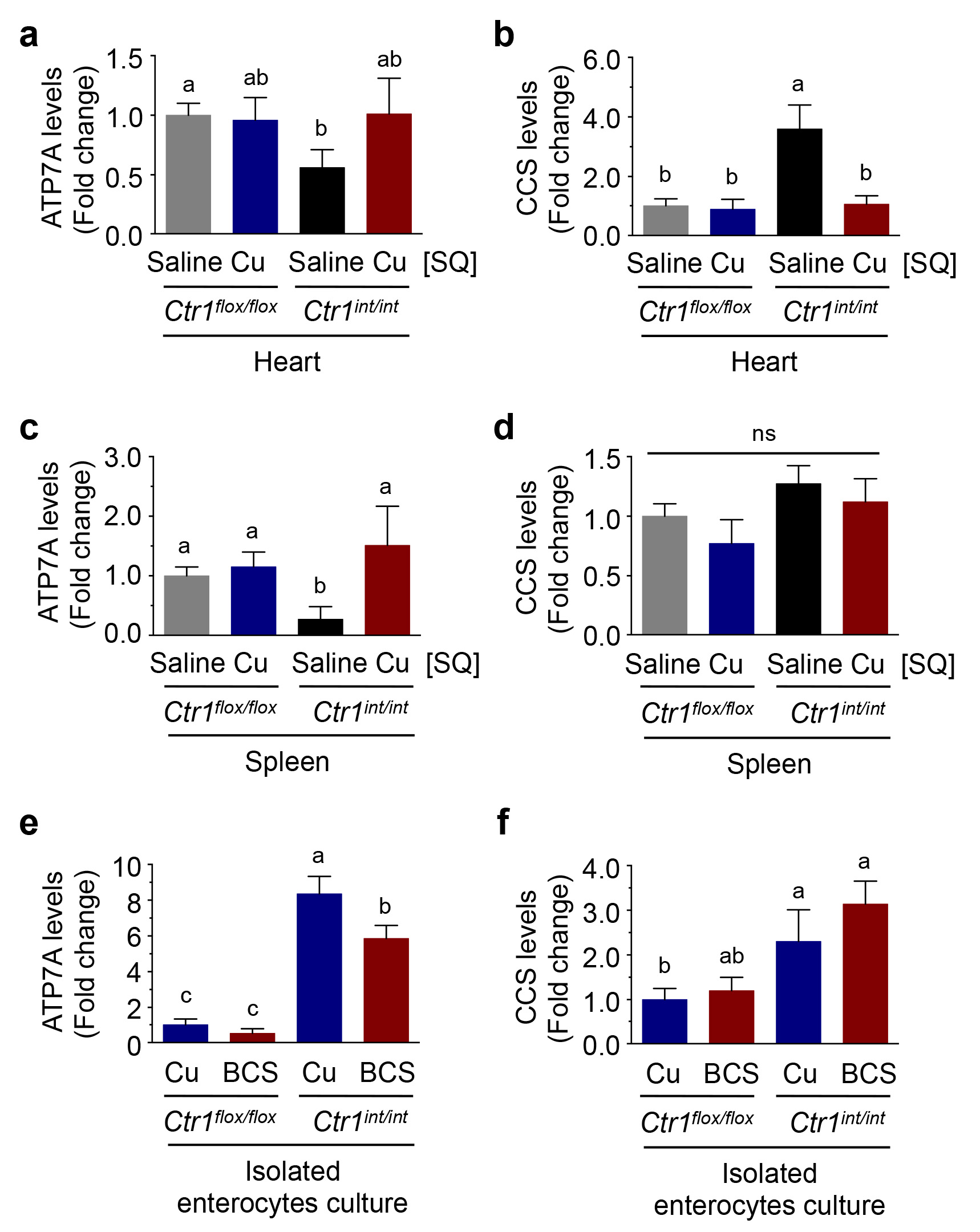
**

**Supplementary Figure 6. Quantification of ATP7A and CCS expression in the heart and spleen from *Ctr1^flox/flox^* and *Ctr1^int/int^* mice administered with saline or Cu and isolated enterocytes culture from *Ctr1^flox/flox^* and *Ctr1^int/int^* mice.** Relative protein abundances of ATP7A (a, c, and e) and CCS (b, d, and f) were quantified by analyzing immunoblots of each tissue from mice (heart, n = 6, 6, 4, and 4; spleen, n = 6, 4, 9, and 4; isolated enterocytes culture, n = 5, 5, 7, and 7) for each condition for statistical analysis. Error bars represent average ± SD, and means indicated with different letters are significantly different from each other at *p*=0.05. ns, not significant (Two-way ANOVA, Tukey’s post hoc test).

**
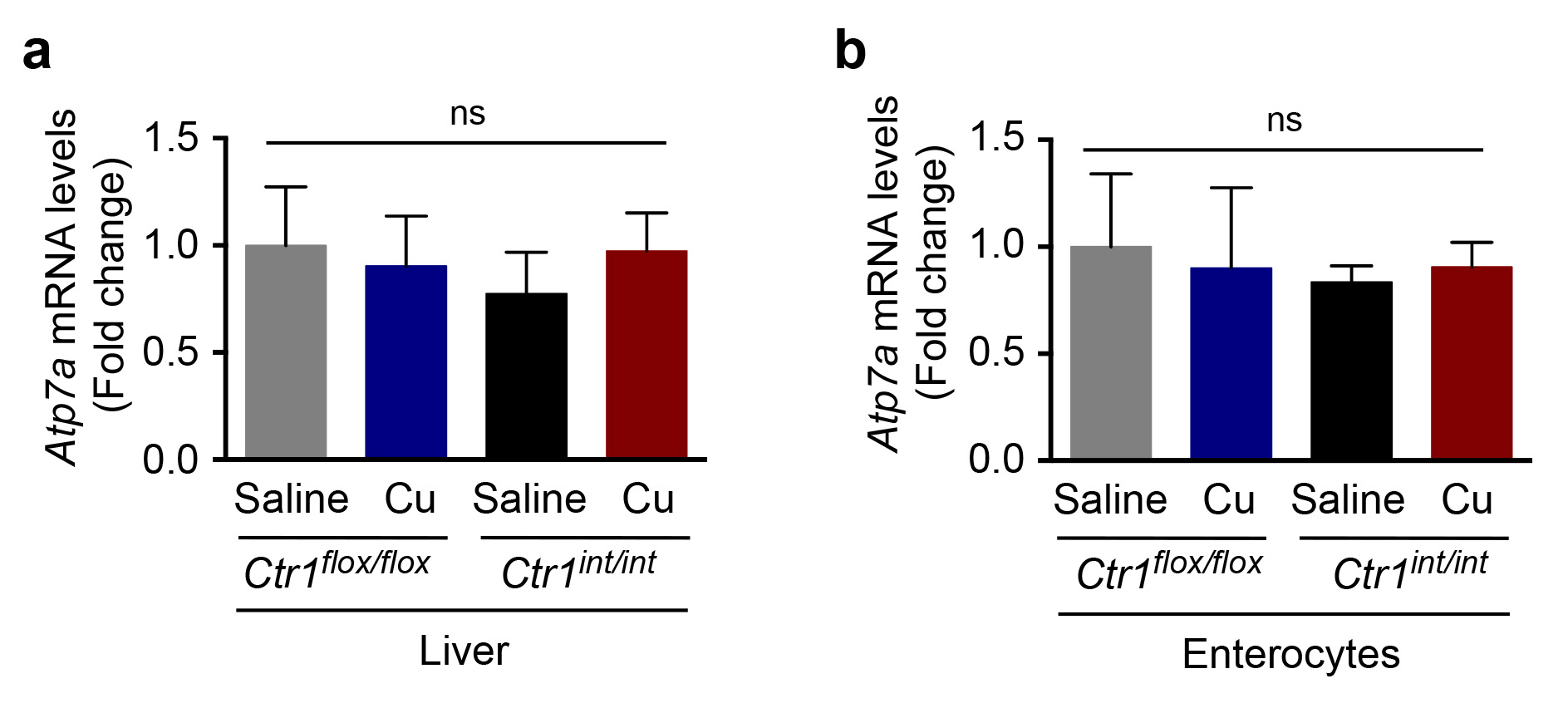
**

**Supplementary Figure 7. Reverse transcription quantitative PCR (RT-qPCR) analysis of *Atp7a* mRNA levels in the liver and intestine of *Ctr1^flox/flox^* and *Ctr1^int/int^* mice SQ administered saline or Cu.** Levels of *Atp7a* mRNA relative to *Gapdh* mRNA levels in the livers (a) and intestinal epithelial cells (b) from the *Ctr1^flox/flox^* and *Ctr1^int/int^* mice two days following SQ administration of saline or 10 µg of Cu-histidine per body weight (g) at P10 were determined in male (n = 2, 3, 3, and 2) and female (n = 4, 2, 3, and 3) mice. Bars indicate mean ± SD of five to six mice for each condition. ns, not significant indicates *p*>0.05 (two-way ANOVA, Tukey’s post hoc test).

**
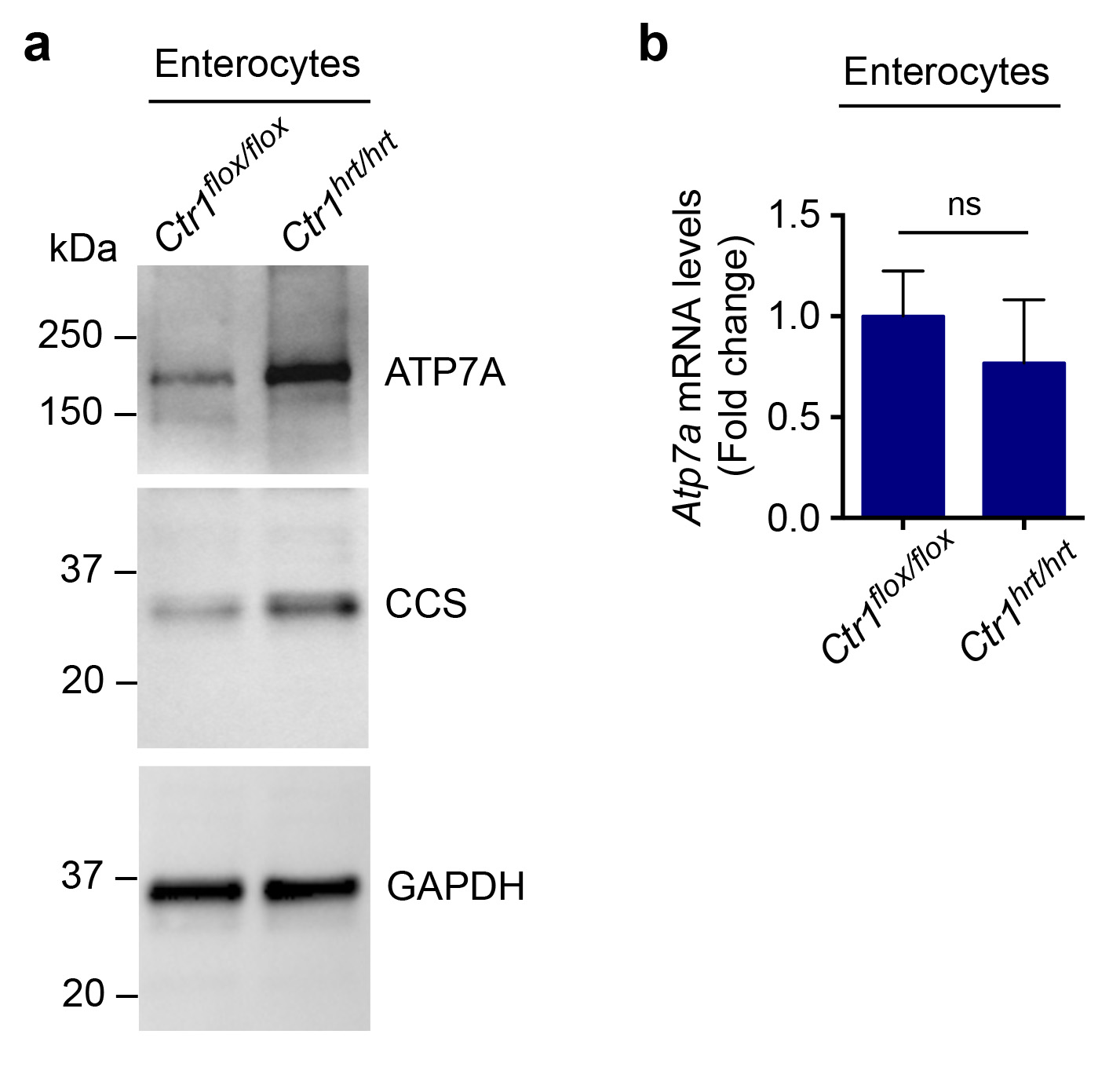
**

**Supplementary Figure 8. Protein and mRNA levels of intestinal ATP7A in cardiac-specific Ctr1 knock-out mice.** Protein abundances (a) and mRNA levels (b) of ATP7A in intestinal cells in *Ctr1^hrt/hrt^* and *Ctr1^flox/flox^* mice were measured by immunoblot analysis and RT-qPCR. A representative result of three independent immunoblots for ATP7A is shown in (a). For RT-qPCR, *Atp7a* and *Gapdh* mRNA levels from five mice (male, 2 and 3; female, 3 and 2) for each condition were determined. Error bars indicate mean ± SD. Statistics: two-tailed unpaired Student’s t-test (ns, *P*>0.05).


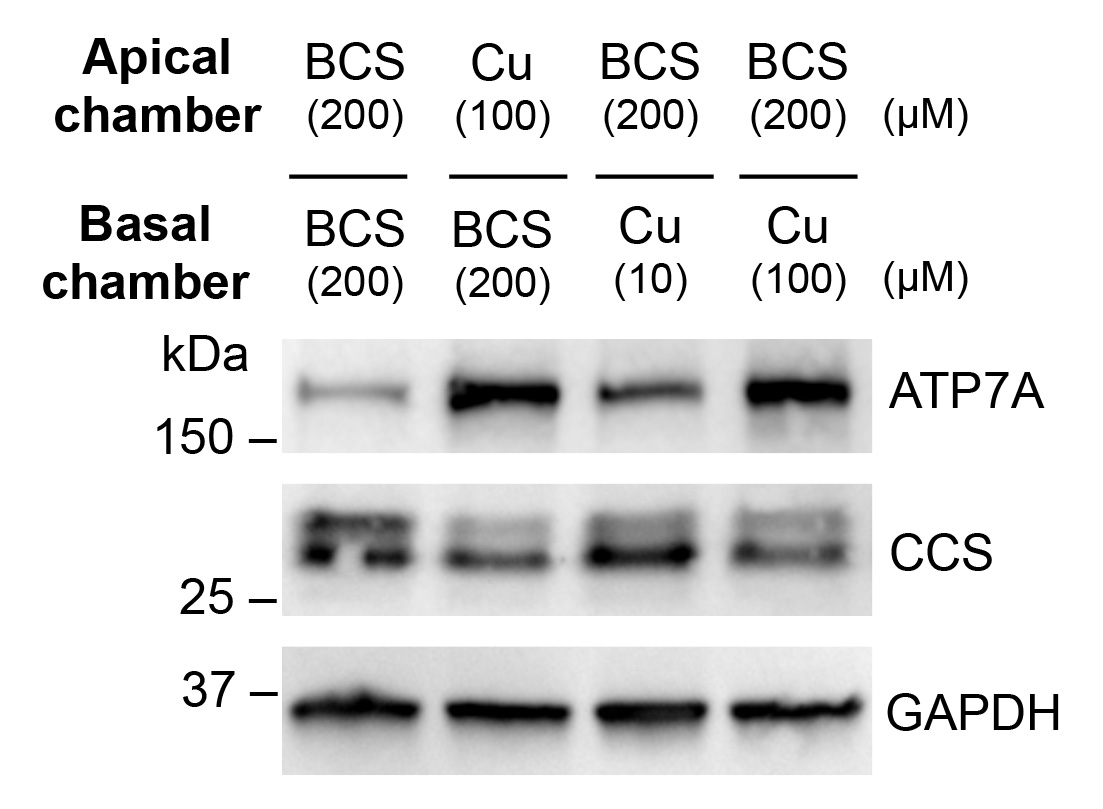


**Supplementary Figure 9. ATP7A protein levels in polarized IEC-6 cells treated with Cu or BCS.** IEC-6 cells were grown on trans-well culture systems until they had formed tight junctions as a monolayer as described in Methods. Cells were treated with Cu or BCS for 12 h on either the apical or basolateral side of IEC-6 cells and cell extracts were then probed with anti-ATP7A, anti-CCS and anti-GAPDH antibodies. Shown is an immunoblot representative of two independent experiments using the indicated antibodies. Full-length blots are presented in Supplementary Figure 17.

**
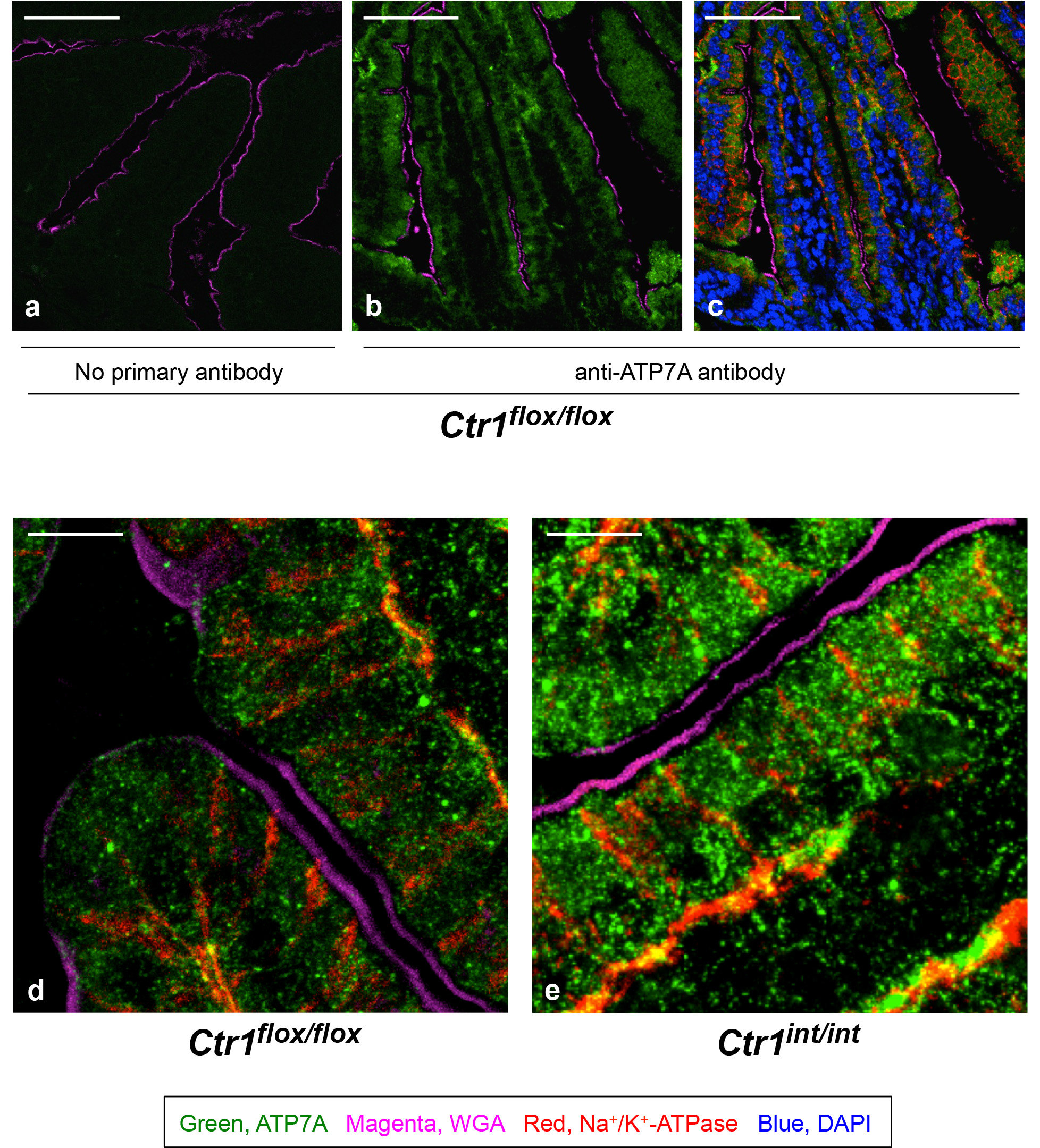
**

**Supplementary Figure 10. Confocal microscopy** **analysis of endogenous ATP7A in the jejunum from *Ctr1^flox/flox^* and *Ctr1^int/int^* mice.** Sections of upper jejunum from *Ctr1^flox/flox^* (a-d) and *Ctr1^int/int^* mice (e) were subjected to confocal immunofluorescence microscopy analysis. Image (a) was obtained without adding anti-ATP7A primary antibody. Images are representative of five independent experiments. Note that b and c are identical images showing different markers. Green, anti-ATP7A; anti-Wheat Germ Agglutinin (WGA, 15 µg/mL); red, anti-Na^+^/K^+^-ATPase alpha-1 subunit; magenta, blue, DAPI. Scale bars, 60 μm (a-c) and 20 μm (d and e).

**
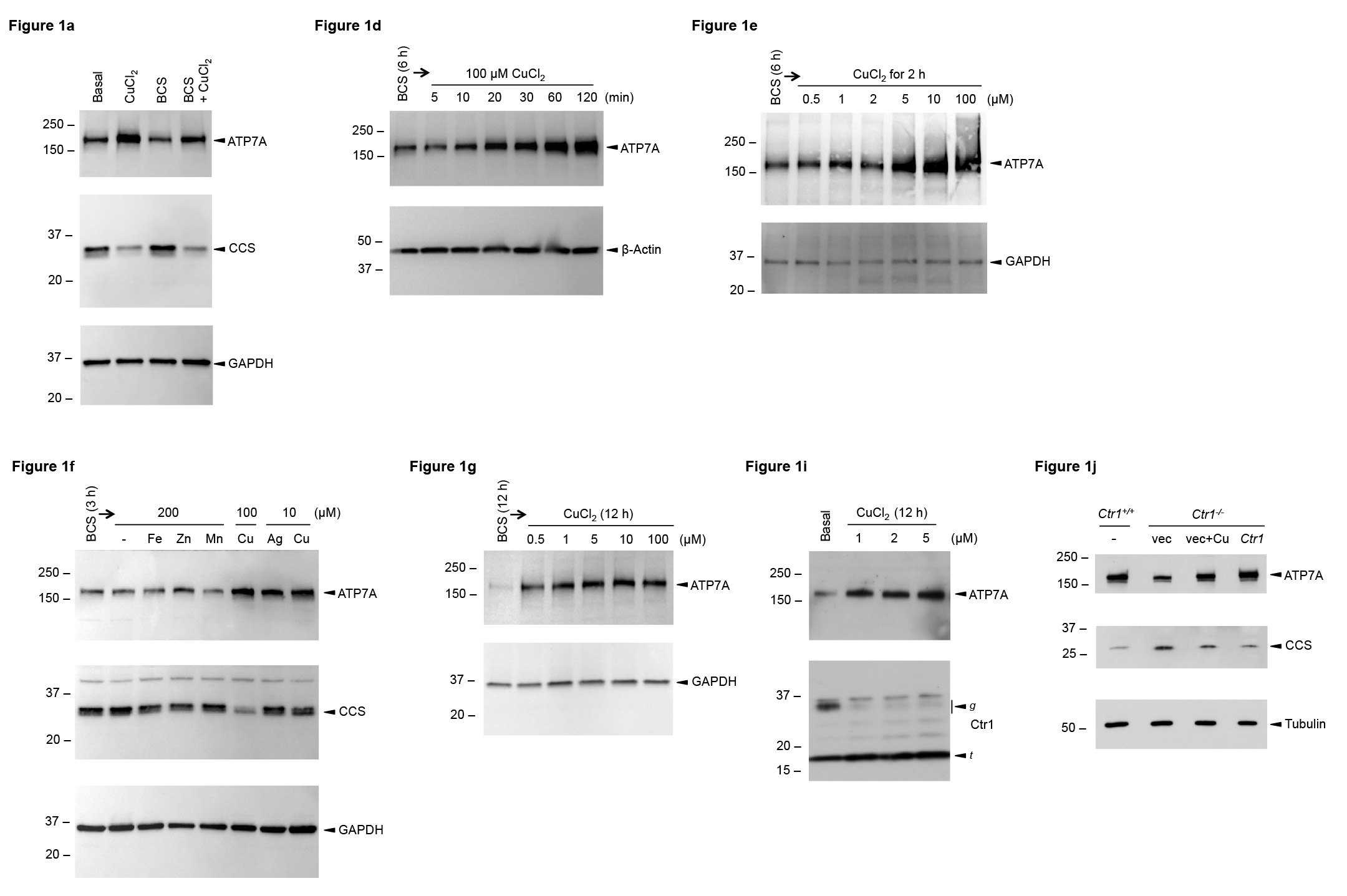
**

**Supplementary Figure 11. Full-length immunoblot images in Figure 1.**

**
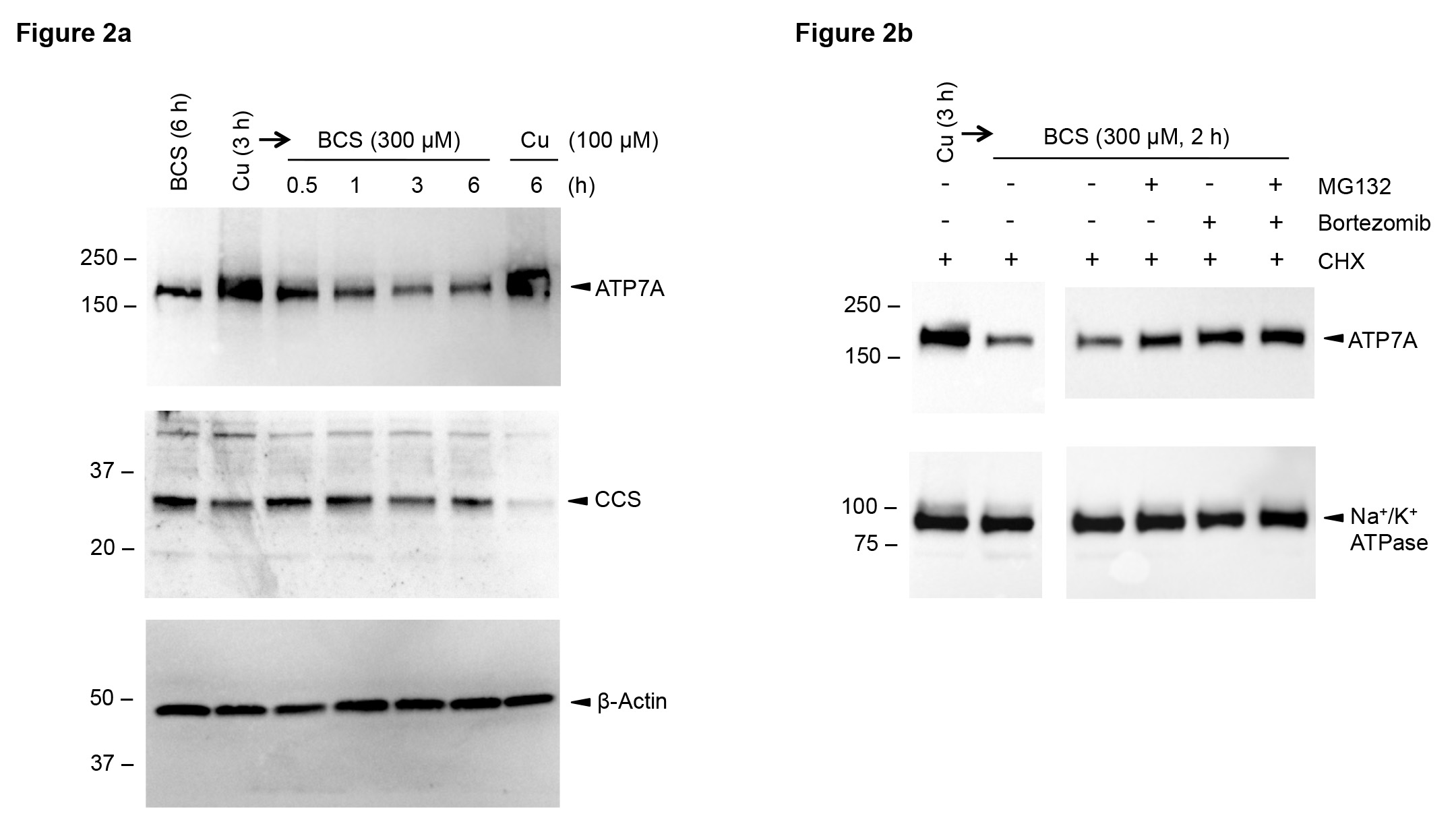
**

**Supplementary Figure 12. Full-length immunoblot images in Figure 2.**

**
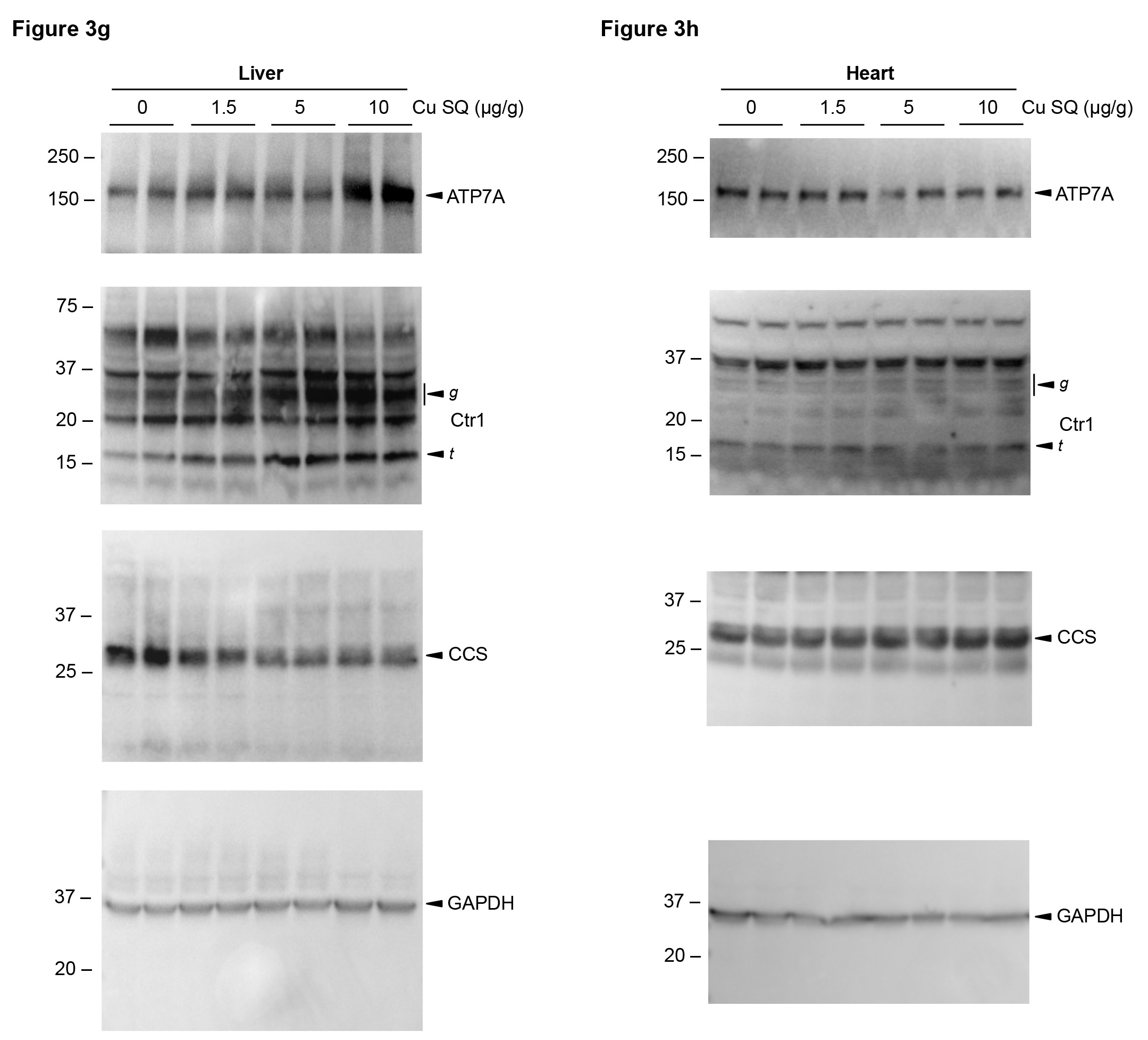
**

**Supplementary Figure 13. Full-length immunoblot images in Figure 3.**

**
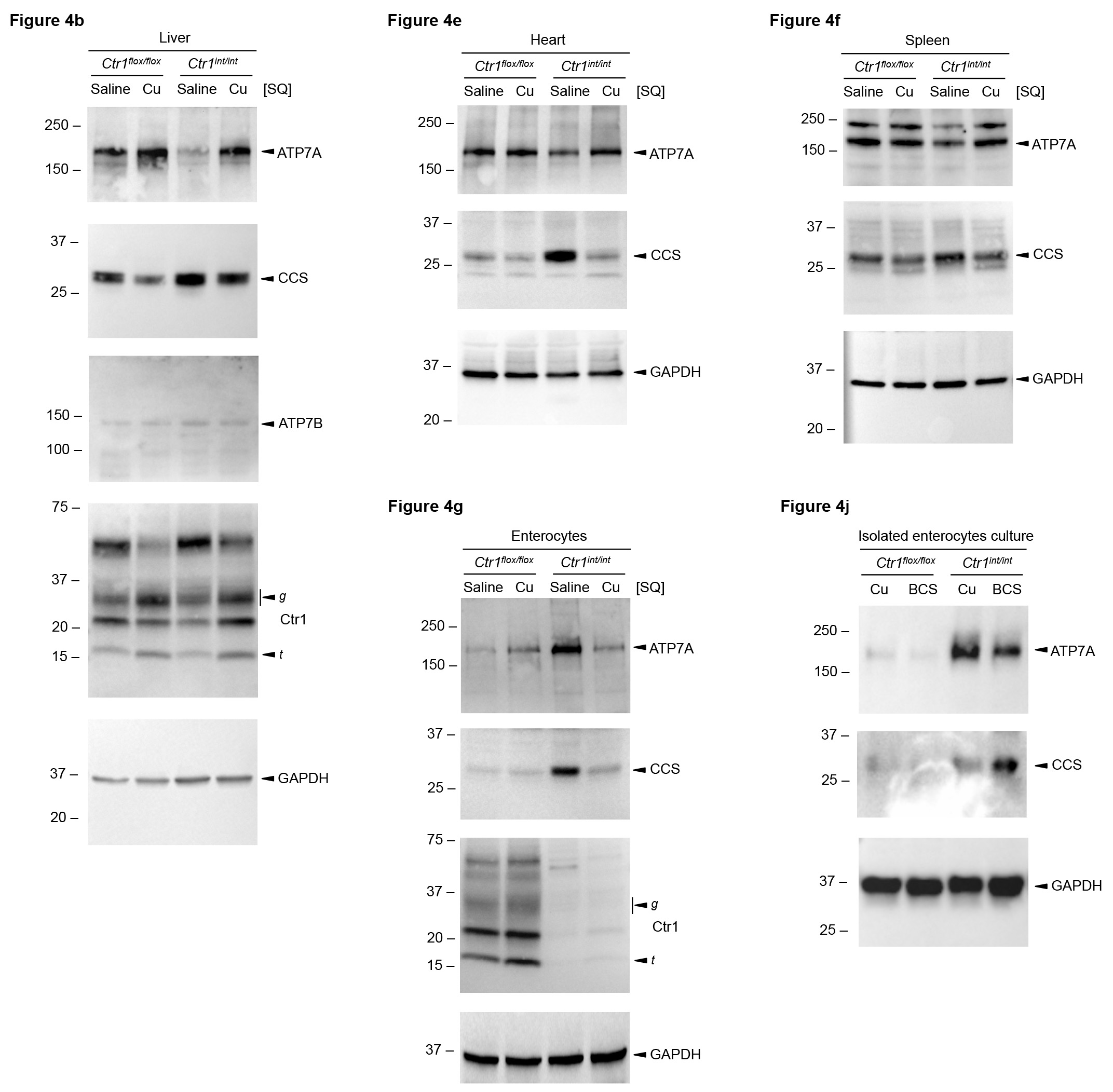
**

**Supplementary Figure 14. Full-length immunoblot images in Figure 4.**

**
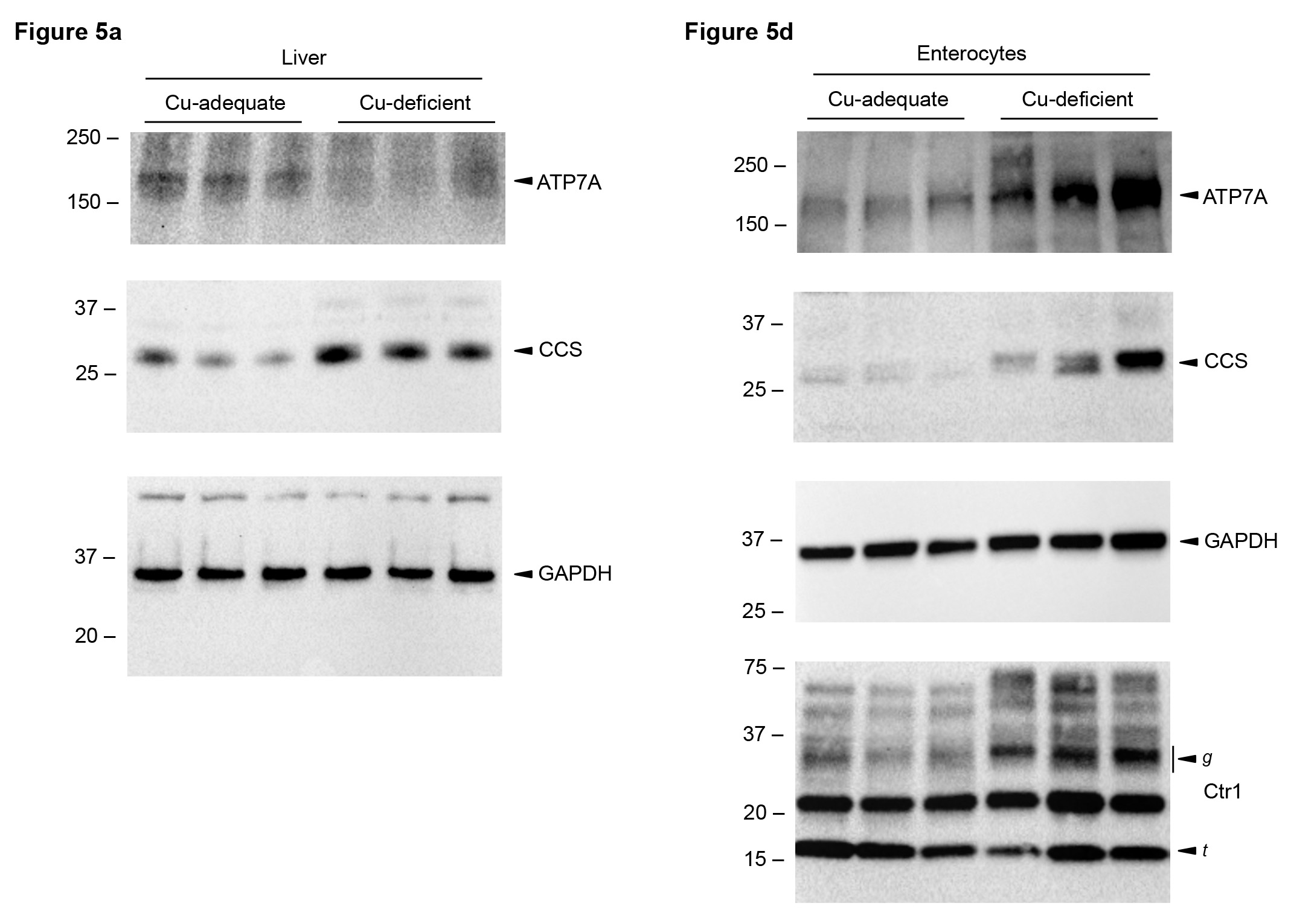
**

**Supplementary Figure 15. Full-length immunoblot images in Figure 5.**

**
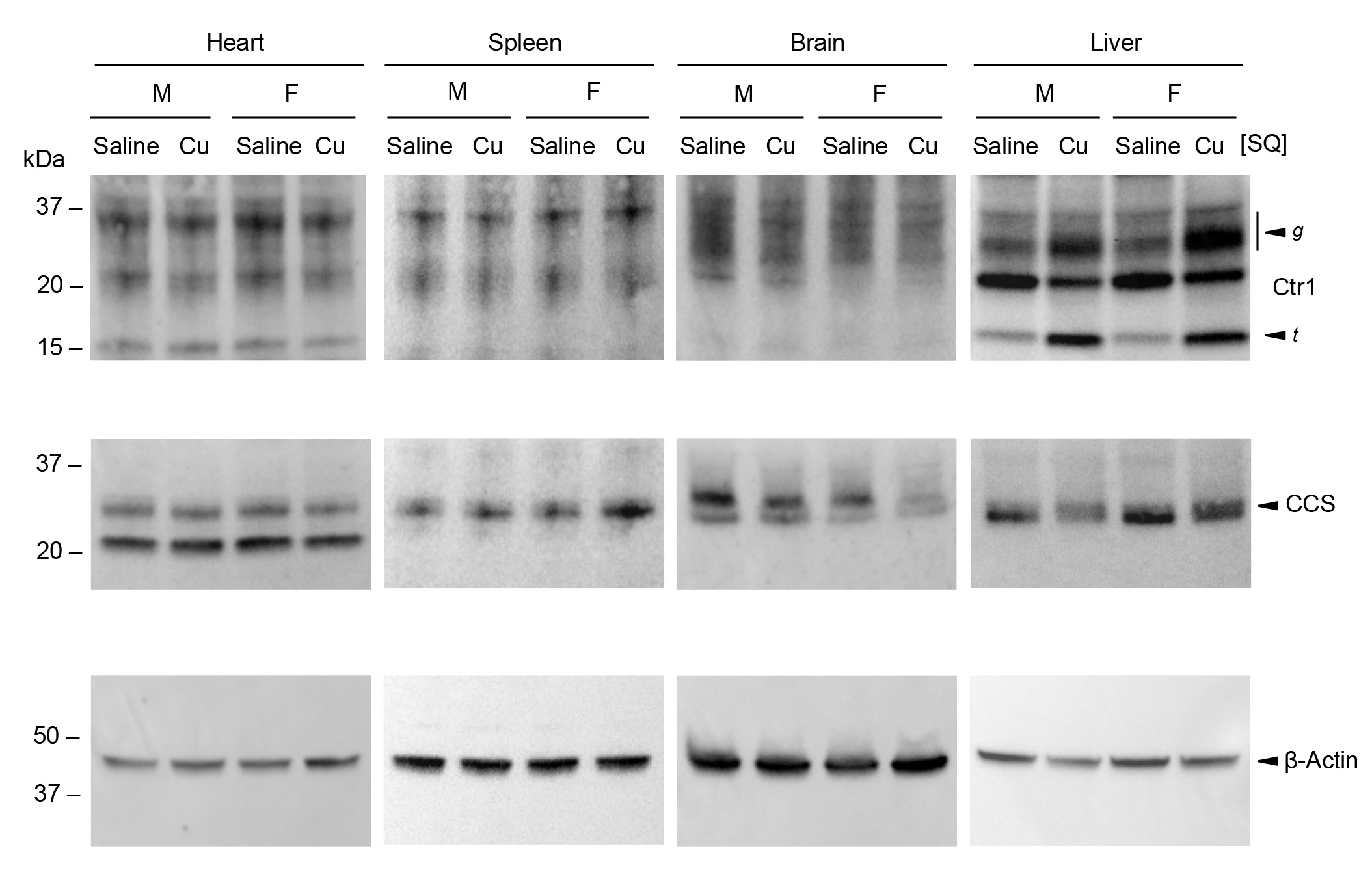
**

**Supplementary Figure 16. Full-length immunoblot images in Supplementary Figure 3.**

**
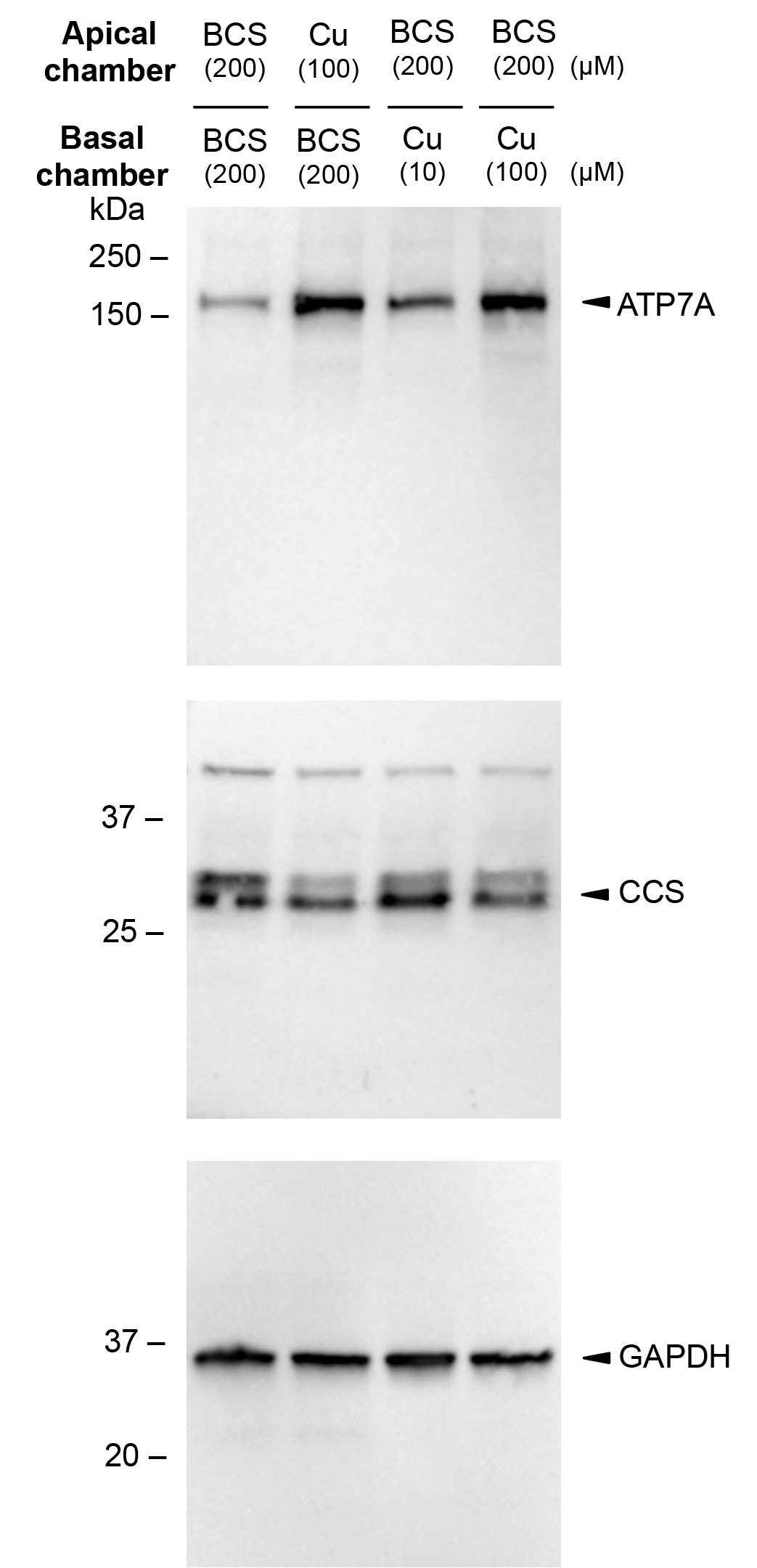
**

**Supplementary Figure 17. Full-length immunoblot images in Supplementary Figure 9.**

**References**

1 Wang, Y., Zhu, S., Weisman, G. A., Gitlin, J. D. & Petris, M. J. Conditional knockout of the Menkes disease copper transporter demonstrates its critical role in embryogenesis. *PLoS One* **7**, e43039, doi:10.1371/journal.pone.0043039 (2012).
